# CRISPR-HAWK: haplotype- and variant-aware guide design toolkit for CRISPR-Cas

**DOI:** 10.1093/bioinformatics/btag708

**Published:** 2026-10-14

**Authors:** Alisa Kumbara, Manuel Tognon, Giulia Carone, Arianna Fontanesi, Nicola Bombieri, Rosalba Giugno, Luca Pinello

**Affiliations:** Department of Engineering for Innovation Medicine, University of Verona, Verona, VR 37134, Italy; Department of Computer Science, University of Verona, Verona, VR 37134, Italy; Department of Computer Science, University of Verona, Verona, VR 37134, Italy; Department of Computer Science, University of Verona, Verona, VR 37134, Italy; Department of Engineering for Innovation Medicine, University of Verona, Verona, VR 37134, Italy; Department of Computer Science, University of Verona, Verona, VR 37134, Italy; Molecular Pathology Unit, Center for Computational and Integrative Biology and Center for Cancer Research, Massachusetts General Hospital, Charlestown, MA 02129, United States; Department of Pathology, Harvard Medical School, Boston, MA 02115, United States; Broad Institute of MIT and Harvard, Cambridge, MA 02142, United States

## Abstract

**Motivation:**

Current CRISPR guide RNA design tools rely on reference genomes, overlooking how genetic variation impacts editing outcomes. As genome editing advances toward clinical applications, incorporating population diversity becomes essential for ensuring therapeutic efficacy across diverse populations.

**Results:**

We present CRISPR-HAWK, a framework integrating individual- and population-scale variants and haplotypes into gRNA design. Analyzing therapeutic targets across 79 648 genomes reveals that genetic variants substantially alter guide performance. For the clinically approved sickle cell disease therapeutic guide targeting BCL11A, we identify haplotypes that completely abolish predicted cutting activity. Across seven therapeutic loci, 82.5% of guides contain variants modifying on-target activity. Variants also create novel protospacer adjacent motif sites generating individual-specific guides invisible to reference-based design. These findings demonstrate that variant-aware selection is critical for equitable genome editing.

**Availability:**

CRISPR-HAWK is available at https://github.com/pinellolab/CRISPR-HAWK and https://github.com/InfOmics/CRISPR-HAWK.

## 1 Introduction

CRISPR-Cas systems present unprecedented opportunities for therapeutic developments by offering a powerful means to precisely modify DNA within living cells. Originally repurposed from Streptococcus pyogenes Cas9 (SpCas9) for targeted genome editing, CRISPR has since evolved into a versatile platform comprising a diverse array of Cas orthologs and engineered variants (Jiang and Doudna 2017). Central to this technology is a guide RNA (gRNA) that directs the Cas complex to a complementary genomic sequence, contingent on the presence of a protospacer adjacent motif (PAM). This modular mechanism enables a spectrum of biological outcomes, including the introduction of double-strand breaks, precise nucleotide substitution via base editors (Rees and Liu 2018), combinations of genetic modifications via prime editing (Anzalone *et al.* 2019), and epigenetic or transcriptional modulation through CRISPRa/I systems (Thakore *et al.* 2016, Kampmann 2018).

The success of CRISPR genome editing relies on two key factors: achieving high on-target efficiency while minimizing off-target activity (Clement *et al.* 2020). On-target efficiency refers to the ability of a gRNA to precisely direct the Cas nuclease to the intended genomic locus, thereby executing the desired genetic modification. Conversely, off-target effects occur when the CRISPR system binds and edits unintended genomic loci with partial sequence similarity, potentially resulting in harmful or confounding mutations (Cho *et al.* 2014). Several computational tools have been developed to support gRNA design by optimizing both aspects (Hanna and Doench 2020). Tools such as Cas-Designer (Cho *et al.* 2014) or CHOPCHOP (Labun *et al.* 2019) identify candidate guides by aligning input PAM sequences against a reference genome. For each reported gRNA, they compute different sequence-based features, such as GC content, and estimate the number and location of potential off-target sites. This information is then used to rank guides based on predicted editing efficiency and target specificity. CRISPick (Doench *et al.* 2016, DeWeirdt *et al.* 2021) and CRISPRon (Anthon *et al.* 2022) integrate machine learning models trained on experimental data to prioritize guides with high predicted activity and minimal off-target potential. CRISPOR (Concordet and Haeussler 2018), meanwhile, integrates information on single nucleotide polymorphisms (SNPs) that may overlap with candidate gRNA sequences.

However, existing tools have a critical limitation: gRNA selection is based on the reference genome, thereby potentially overlooking on-target sites introduced or modulated by genetic variants. While CRISPOR annotates candidate gRNAs with overlapping SNPs, the guide selection process still relies on the reference sequence. Consequently, the combined effect of multiple variants or haplotypes is not considered, nor is the impact of genetic variants on on-target activity quantitatively assessed. While it is well recognized that variants can create or modulate off-target sites (Cancellieri *et al.* 2023, Lazzarotto *et al.* 2025), there is growing evidence indicating that variants can affect on-target efficiency. This occurs through direct alteration of the intended protospacer sequences or changes in its local genomic context (Canver *et al.* 2018). Such effects are critical in therapeutic applications, where even subtle differences in editing efficiency or specificity can impact clinical efficacy and safety (Lessard *et al.* 2017, Liu *et al.* 2021).

The clinical significance of this problem is exemplified by recent therapeutic applications. Casgevy (exagamglogene autotemcel), one of the first FDA-approved CRISPR therapies for sickle cell disease and beta-thalassemia, relies on precise editing of the BCL11A enhancer using guide sg1617 (Frangoul *et al.* 2021). Yet these treatments, designed using reference genomes, may have variable efficacy across genetically diverse populations.

As genome editing advances toward personalized and clinical applications, the need to account for both individual- and population-level genetic variation becomes increasingly important (Scott and Zhang 2017). Incorporating genetic diversity into gRNA design is key to enhancing on-target efficiency, reducing off-target effects, and ensuring the safety and efficacy of therapeutical outcomes. To address these challenges, we developed CRISPR-HAWK, representing, to our knowledge, the first framework to integrate both genetic variants and haplotypes in gRNA design. By reconstructing individual haplotypes from genetic population-scale variant datasets, CRISPR-HAWK enables sample-specific guide selection while providing comprehensive assessment of guide performance. On-target efficiency of candidate gRNAs is predicted using machine learning models, while off-target activity is assessed using CRISPRitz (Cancellieri *et al.* 2020), a robust off-target nomination search engine. Candidate guides are further annotated with genetic variants, and functional information to support informed gRNA selection. We demonstrate the utility of CRISPR-HAWK, by designing candidate gRNAs targeting clinically relevant or widely tested genomic regions in human, while accounting for genetic variants from the 1000 Genomes Project (1000G) (1000 Genomes Project Consortium *et al.* 2015, Zheng-Bradley *et al.* 2017), Human Genome Diversity Project (HGDP) (Bergström *et al.* 2020), and Genome Aggregation Database (gnomAD) (Karczewski *et al.* 2020, Chen *et al.* 2024) datasets. We show that genetic variation gives rise to numerous population- and individual-specific alternative gRNAs within each analyzed target region, revealing that many candidate guides are overlooked when relying solely on the reference genome. Moreover, we analyze how genetic variants impact on-target activity of gRNAs designed on the reference genome. These findings underscore the importance of adopting variant-aware strategies to ensure accurate, efficient, and equitable genome editing, particularly in therapeutic contexts.

## 2 Methods

CRISPR-HAWK is a command-line tool designed for the efficient enumeration, scoring, and annotation of CRISPR-Cas guide RNAs within user-defined genomic regions. Figures 1 and 2 provide an overview of its architecture, illustrating the required and optional inputs, the main computational steps, and the structure of the resulting outputs. The following subsections detail the implementation of CRISPR-HAWK, including its strategy for integrating genetic variants and haplotype data, the algorithmic details underlying gRNA search and scoring, and the annotation pipeline. We further describe the datasets and experimental settings used for benchmarking and performance evaluation.

**Figure 1:**
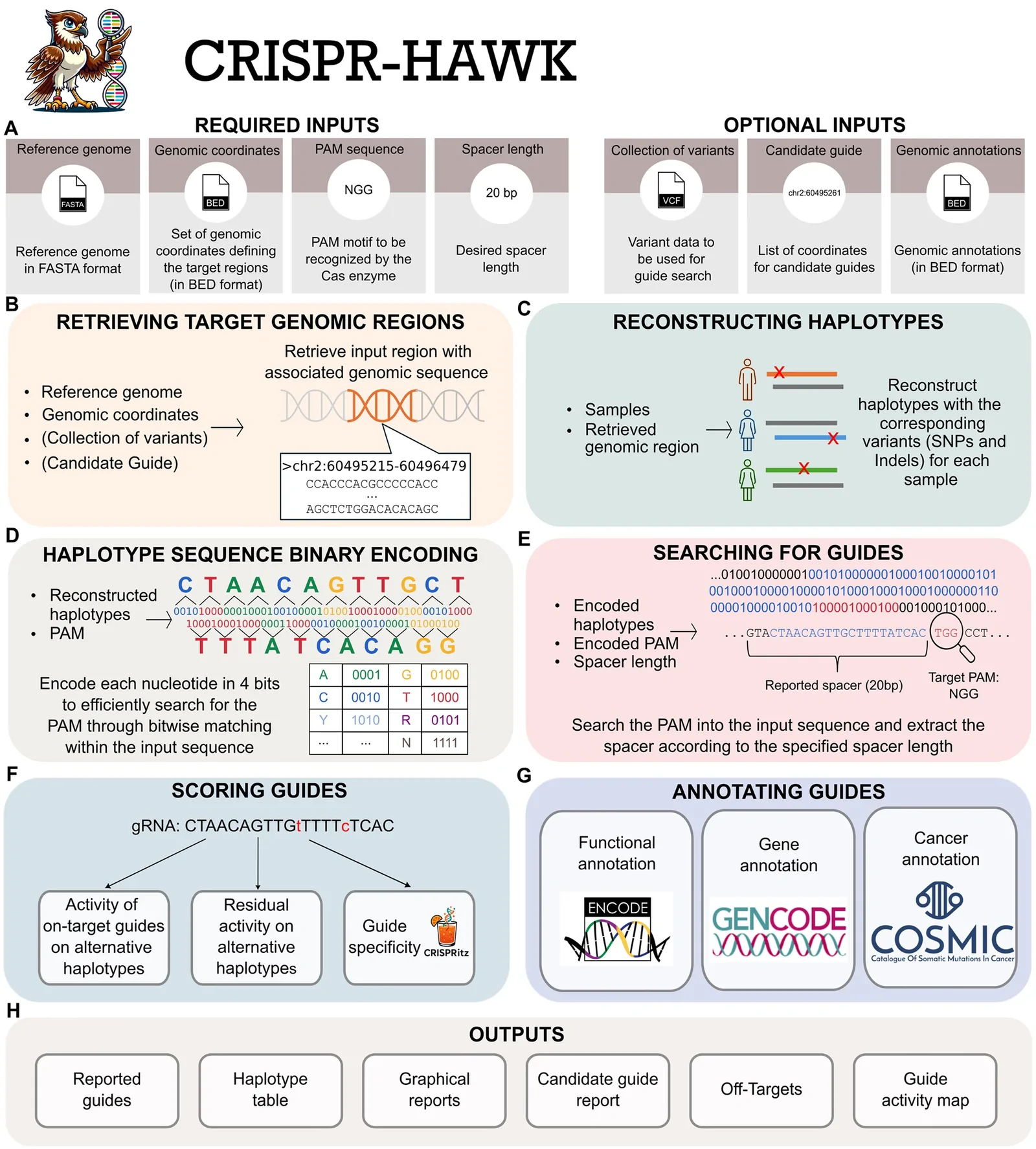
Overview of CRISPR-HAWK architecture. (A) CRISPR-HAWK designs gRNAs from a reference genome, by specifying genomic coordinates, a PAM motif, and a spacer length. Optional inputs include variant datasets for variant- and haplotype-aware guide design, genomic annotation files, and user-provided candidate guide coordinates for assessing variant effects. (B) Genomic regions of interest are extracted and (C) combined with variant data, when available, to reconstruct haplotype-resolved sequences. (D) Reconstructed haplotypes and PAM sequences are encoded using a 4-bit binary scheme that supports IUPAC ambiguity codes, enabling fast bitwise pattern matching. (E) Encoded sequences are scanned to identify PAM occurrences, and adjacent spacers are extracted to generate candidate gRNAs. (F) Candidate guides are evaluated using three complementary metrics: on-target efficiency of haplotype-matched guides, residual on-target activity of reference-designed guides on variant-containing target sequences, and guide specificity via existing predictive models. (G) Guides are annotated with functional, gene-level, and cancer-related features using default annotation tracks (ENCODE, GENCODE, COSMIC) or user-provided BED files. (H) CRISPR-HAWK generates comprehensive outputs including a table of all candidate guides identified within the input region. Optional outputs include a table of reconstructed haplotypes, graphical reports, a dedicated report for user-selected candidate guides, a genome-wide list of predicted off-target sites, and a guide activity map illustrating residual on-target activity with specificity.

**Figure 2:**
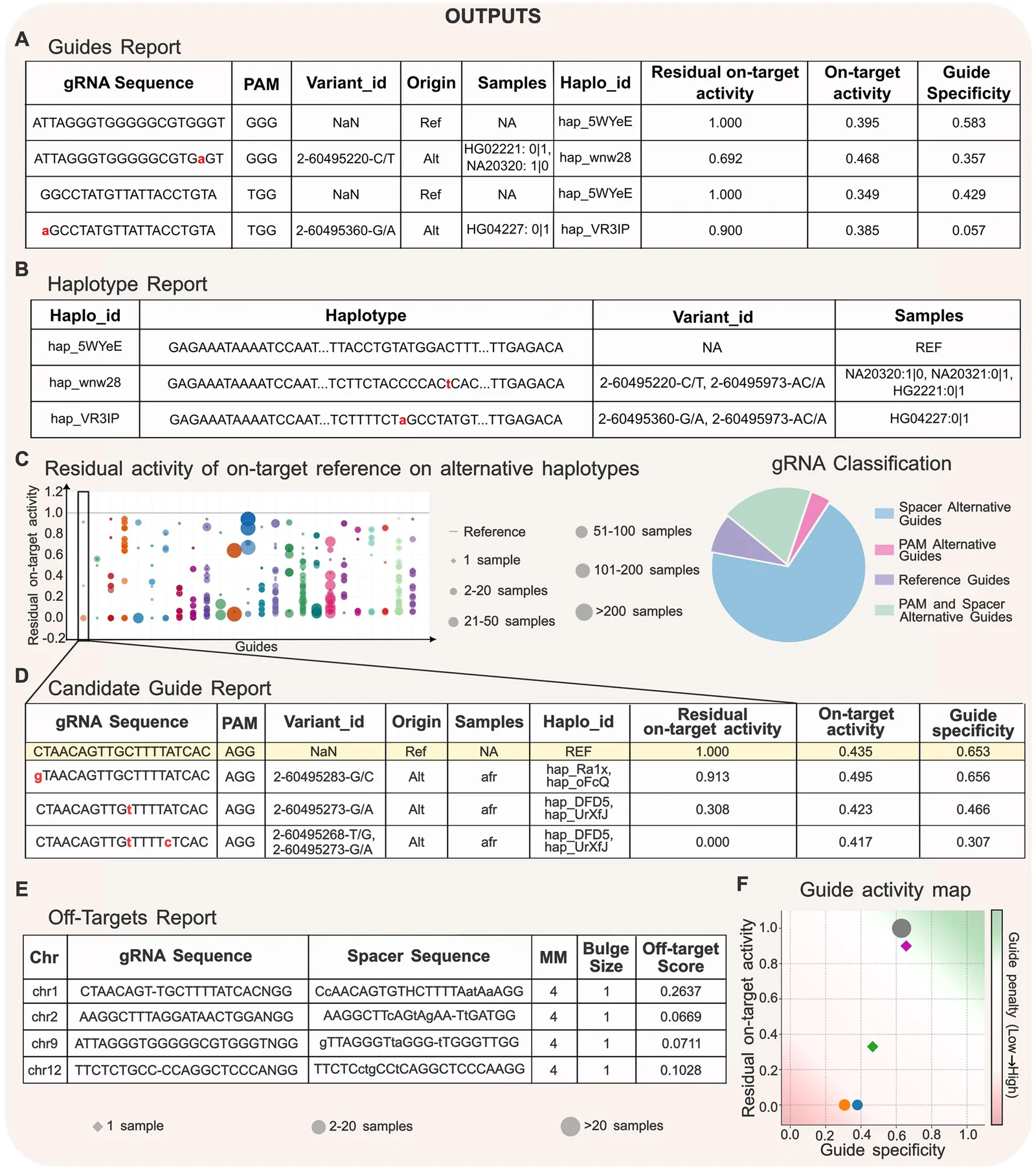
Overview of CRISPR-HAWK outputs and visualizations. (A) The primary output summarizes all candidate gRNAs with their associated performance metrics in a tabular report, including on-target efficiency, residual on-target activity, and guide specificity. (B) When requested, a haplotype table is provided, listing each reconstructed haplotype alongside the corresponding samples and variants. (C) Graphical reports include scatter plots depicting residual on-target activity across variant-containing target sequences, with point size indicating the number of individuals carrying each variant, and pie charts classifying gRNAs by type (reference, spacer alternative, PAM alternative, or both). (D) Users may optionally restrict the analysis to selected candidate guides. In this mode, the tool produces a dedicated report detailing all variant-containing target sequences and their associated scores for on-target efficiency, residual on-target activity, and guide specificity. (E) A genome-wide list of predicted off-target sites is generated for each candidate gRNA using CRISPRitz. (F) The guide activity map depicts residual on-target activity with guide specificity, providing a global view of guide performance across diverse genomic backgrounds.

### 2.1 Required and optional input parameters

CRISPR-HAWK operates through a set of main input parameters guiding its search and annotation workflow (Fig. 1A). Four inputs are mandatory: (i) a reference genome in FASTA format, (ii) genomic coordinates specifying the target regions in BED format, (iii) the PAM recognized by the selected Cas nuclease, and (iv) the desired spacer length. In addition to these core inputs, users may optionally provide variant datasets in VCF format (phased or unphased) to enable variant- and haplotype-aware guide design, as well as pre-defined candidate gRNAs for focused evaluation. Functional genomic annotation in BED format can also be provided to enrich the resulting guides with contextual information such as regulatory or coding region overlap.

### 2.2 Retrieving target genomic regions and haplotypes reconstruction

Given a reference genome and a set of genomic coordinates, CRISPR-HAWK begins by extracting the corresponding target genomic regions from the reference genome (Fig. 1B). To ensure sufficient context for both gRNA identification and downstream analyses, such as on-target efficiency prediction and variant mapping, each region is symmetrically extended by 100 base pairs upstream and downstream. The resulting extended sequences form the basis for integrating genetic variation and reconstructing haplotype-resolved representations of the target loci (Fig. 1C). To incorporate genetic diversity, CRISPR-HAWK enriches the extracted sequences with single-nucleotide variants (SNVs) and short insertions/deletions (indels) provided in input VCF files. The tool supports both phased and unphased genotype data. For phased data, CRISPR-HAWK reconstructs haplotypes by creating two separate sequences per individual corresponding to the maternal and paternal alleles and sequentially inserts the appropriate variants. This process yields accurate reconstruction of each individual’s diploid genomic context within the target region. In the case of unphased data, where the chromosomal origin of each allele is unknown, heterozygous and multiallelic positions are initially encoded using IUPAC ambiguity codes which represent sets of possible nucleotides at each site. To resolve these ambiguities, CRISPR-HAWK performs combinatorial enumeration of all feasible haplotypes that could arise from the observed genotypes, only for sequences identified as guide candidates (see Section 2.3). This selective strategy significantly reduces memory consumption and computational time, enabling the analysis of large population-scale datasets, such as gnomAD. To further reduce computational burdens, CRISPR-HAWK collapses biologically equivalent haplotypes using hashing-based deduplication. Importantly, the haplotype reconstruction process considers the combined effect of SNVs and indels in both phased and unphased contexts, representing each individual’s sequence consistently. Each reconstructed haplotype is annotated with rich metadata including the list of occurring variants, samples or populations of origin, and phasing status (in case of phased variants only). This haplotype-centric approach enables CRISPR- HAWK to identify gRNAs with improved population coverage and individual-level precision.

### 2.3 Genomic regions binary encoding and search for candidate guides

Guide RNAs are identified by scanning the reconstructed haplotype sequences for the user-specified PAM motif on both forward and reverse strands. This step is computationally intensive, as it requires exhaustive base-by-base comparisons across potentially thousands of haplotypes. To address this, CRISPR-HAWK employs an optimized binary encoding strategy for guide discovery (Fig. 1D). Each haplotype is converted into a compact 4-bits-per-base representation that supports both standard and IUPAC ambiguity codes. PAM detection is then performed via bitwise AND operations rather than character-level string comparisons. This approach reduces the constant-factor cost of the per-position matching step while decreasing the memory footprint required to store haplotype sequences relative to standard ASCII representation. Both the binary-encoded implementation and a naïve character-based scan share the same asymptotic time complexity O(H·L), where H denotes the number of haplotypes and L their length. The advantage of the binary representation therefore lies in improved constant-factor runtime and reduced memory footprint, which becomes increasingly important when analyzing large genomic regions or genetically diverse populations (see Supplementary Data Section 1). Upon detection of a valid PAM sequence, the adjacent spacer sequence, of user-defined length, is extracted (Fig. 1E). For hits on the reverse strand the corresponding sequences are reverse-complemented to preserve consistent orientation. These extracted sequences are hereafter referred to as candidate gRNAs. To capture the impact of genetic variation, CRISPR-HAWK systematically flags all candidate gRNAs that overlap with variant sites. Within each guide, variant-affected nucleotides are displayed in lowercase, while reference-matching bases remain in uppercase, enabling clear distinction between conserved and variable positions. This representation facilitates rapid visual inspection and supports flexible downstream filtering, allowing users to prioritize guides based on their tolerance or sensitivity to genetic variation, depending on the intended application.

### 2.4 Scoring and annotating guides

Accurate evaluation of the designed gRNAs requires assessing their cleavage efficiency, specificity, and functional genomic context. CRISPR-HAWK leverages multiple scoring models (Fig. 1F) and a comprehensive annotation framework (Fig. 1G) to support informed guide selection.

We distinguish three complementary performance metrics. On-target efficiency predicts the cleavage activity of a guide at its intended target site, estimated using sequence-based machine learning models. Residual on-target activity predicts how effectively a reference-designed guide will cleave variant-containing target sequences, quantifying the impact of genetic mismatches on expected performance. Guide specificity measures selectivity across the genome by aggregating predicted off-target cleavage likelihoods. The following subsections describe each component in detail.

#### 2.4.1 Predicting on-target efficiency

Several computational models have been developed to predict on-target cleavage efficiency of gRNAs (Doench *et al.* 2014, Sherkatghanad *et al.* 2023). CRISPR-HAWK includes a diverse set of state-of-the-art models to provide robust and complementary estimates of guide activity.

For SpCas9, CRISPR-HAWK incorporates five scoring methods: Azimuth (Doench *et al.* 2016), Rule Set 3 (RS3) (DeWeirdt *et al.* 2022), CRISPRon (Anthon *et al.* 2022), sgDesigner (Hiranniramol *et al.* 2020), and PLM-CRISPR (Hou *et al.* 2025). Azimuth employs a machine learning model trained on more than 4000 experimentally validated gRNAs targeting coding regions across 17 genes. RS3 extends Azimuth by incorporating additional sequence and thermodynamics features in the model, including poly(T) content, spacer-DNA melting temperature, and minimum free energy of the folded gRNA structure. CRISPRon is a deep learning model trained on over 23 000 gRNAs, combining sequence one-hot encoding with thermodynamic features to estimate on-target activity and prioritize candidate guides. sgDesigner, instead, was trained on a custom plasmid library comprising more than 7400 sgRNAs. It employs a stacked generalization strategy integrating support vector machine and XGBoost models through logistic regression, leveraging both sequence and structural features. PLM-CRISPR is a dual-input deep learning framework that models sgRNA sequences efficiency on different Cas9 protein variants. While predicting gRNA activity, it accounts for sequence-protein interactions.

In addition, to support Cas12a applications, CRISPR-HAWK includes DeepCpf1 (Kim *et al.* 2018), a deep learning model trained to estimate the on-target activity of Cpf1 (Cas12a) systems using high-throughput experimental profiling data.

Although these models all predict on-target activity and show moderate-to-high agreement, they were developed using different nucleases, guide designs, experimental readouts, training datasets, and machine learning frameworks. Consequently, CRISPR-HAWK reports their scores independently rather than combining them into a single consensus ranking. Detailed guidance on interpreting and selecting among these scores, together with a summary of each method’s nuclease system, PAM compatibility, training dataset, model architecture, and principal limitations, is provided in Supplementary Data Section 2.

#### 2.4.2 Predicting residual on-target activity across variant haplotypes

A critical challenge in variant-aware gRNA design is predicting how genetic variants at target sites affect the cutting efficiency of reference-designed guides. We address this by repurposing established off-target prediction models as surrogates for residual on-target activity when variants create mismatches. The key insight is that the same biophysical principles governing Cas9 binding and cleavage at mismatched off-target sites also apply when variants introduce mismatches at intended on-target sites.

CRISPR-HAWK implements this approach using two established models: cutting frequency determination (CFD) (Doench *et al.* 2016) and Elevation (Listgarten *et al.* 2018). While originally developed to predict off-target cleavage with sequence mismatches, these models quantify the fundamental relationship between sequence complementarity and Cas nuclease activity. When genetic variants alter a target site, they create imperfect base-pairing analogous to off-target mismatches.

For each reference-designed gRNA, CRISPR-HAWK identifies all alternative target sequences arising from genetic variants in the input population. These variant-containing sequences represent individual-specific on-target sites that differ from the reference. The tool then computes CFD and Elevation scores between each reference guide and its alternative targets. Lower scores indicate reduced predicted cleavage efficiency due to variant-induced mismatches, while scores near 1.0 suggest preserved activity despite genetic variation. This framework enables systematic assessment of how population-level genetic diversity may modulate therapeutic efficacy of CRISPR-Cas systems designed from reference genomes.

Among the available off-target scoring functions, CFD and Elevation were adopted for this purpose because both operate directly on guide-mismatched target pairs and expose an explicit, position- and type-dependent mismatch penalty that has been validated in variant-aware contexts (Cancellieri *et al.* 2023), making them directly applicable to residual on-target estimation. Supplementary Data Section 3 provides a comparison with more recent deep learning-based off-target predictors, together with the rationale for this choice.

#### 2.4.3 Estimating guide specificity

To estimate guide specificity, CRISPR-HAWK integrates CRISPRitz (Cancellieri *et al.* 2020), a high-performance genome-wide search engine that identifies putative off-target sites allowing a user-defined number of mismatches and DNA/RNA bulges. For computational efficiency, off-target enumeration is performed against the reference genome, providing a baseline estimate of global specificity for each candidate guide. To extend this analysis to population-scale contexts, CRISPR-HAWK generates input files compatible with CRISPRme (Cancellieri *et al.* 2023), a variant- and haplotype-aware off-target nomination tool. This interoperability enables evaluation of guide specificity across diverse genetic backgrounds.

For each predicted off-target site, CRISPR-HAWK computes CFD and Elevation scores to estimate the likelihood of unintended cleavage. A global specificity score is then calculated for each gRNA by aggregating the CFDs of all its predicted off-targets, allowing users to rank and prioritize guides with minimal off-target potential.

#### 2.4.4 Annotating guides

To support biological interpretation and downstream filtering, CRISPR-HAWK annotates candidate gRNAs with functional genomic context and sequence-derived features. Guides are annotated using user-provided genomic features, such as regulatory regions, coding exons, or disease-associated loci, like COSMIC cancer-related annotations (Sondka *et al.* 2024). Additionally, CRISPR-HAWK computes sequence-derived features that may influence gRNAs performance. GC content is calculated for each guide, as it has been shown to affect Cas binding affinity and gRNA stability (Yuen *et al.* 2017).

### 2.5 Reports generation

CRISPR-HAWK generates a comprehensive set of output files and visualizations summarizing all candidate gRNAs and their associated features (Figs. 1H and 2). The main output is a tabular report listing each candidate gRNA with its genomic coordinates, strand, PAM sequence, spacer sequence, predicted efficiency and specificity scores, functional and gene-level annotations, as well as haplotype and sample associations when variant data are provided (Fig. 2A). When variant-aware analysis is performed, CRISPR-HAWK optionally produces a table reporting the reconstructed haplotypes for the input genomic region, including the constituent variants, population of origin, and allele frequencies (Fig. 2B).

To facilitate data interpretation, the tool provides a series of graphical summaries (Fig. 2C). These include comparative plots of on-target efficiency scores for reference-derived versus alternative guide sequences, illustrating the influence of genetic variation on predicted activity. Additional visualizations show the distribution of identified gRNAs by type (reference-only, variant-containing spacer, novel PAM, or both).

If candidate guide coordinates are supplied, CRISPR-HAWK generates a dedicated report restricted to the specified guides, detailing their corresponding alternative sequences arising from genetic variants, with associated residual on-target activity scores (Fig. 2D). When the off-target search option is enabled, an off-target summary table is produced, listing all predicted off-target sites for each guide with their genomic positions, mismatch and bulge counts, and CFD/Elevation scores (Fig. 2E). For each candidate guide, CRISPR-HAWK also produces a guide activity map that illustrates residual on-target activity across variant haplotypes with guide specificity, providing a global view of guide performance across diverse genomic backgrounds (Fig. 2F).

## 3 Results

We evaluated CRISPR-HAWK across clinically relevant and well-characterized genomic regions to assess its ability to design and characterize variant- and haplotype-aware gRNAs using population-scale variant datasets. All analyses were performed using the GRCh38 human genome assembly, enriched with variants from three major population-scale datasets: the 1000 Genomes Project (1000G) (1000 Genomes Project Consortium *et al.* 2015, Zheng-Bradley *et al.* 2017), the Human Genome Diversity Project (HGDP) (Bergström *et al.* 2020), and the Genome Aggregation Database (gnomAD) (Karczewski *et al.* 2020). The 1000G dataset includes whole-genome sequencing data from 2504 individuals spanning 26 populations, organized into five superpopulations. The HGDP dataset comprises 929 individuals representing 54 populations, grouped into broader ancestries. The gnomAD dataset aggregates high-quality whole-genome sequencing data from 76 215 unrelated individuals, encompassing diverse genetic ancestries.

Using these datasets, we applied CRISPR-HAWK to two complementary tasks. First, we performed de novo guide enumeration and efficiency prediction at the BCL11A +58 erythroid enhancer, the target of sg1617, the clinically optimized guide used in Casgevy (exagamglogene autotemcel), one of the first FDA-approved CRISPR therapies for sickle cell disease and β-thalassemia. This analysis enabled both identification of alternative candidate guides and assessment of how population-level variants affect the predicted activity of sg1617 itself. Second, we quantified the effects of genetic variation on residual on-target activity and guide specificity across a broader panel of guides, including therapeutic gRNAs currently in clinical development and benchmark guides commonly used in off-target characterization studies.

To evaluate the computational scalability of CRISPR-HAWK, we benchmarked it evaluating the impact of key parameters on runtime and memory usage. Specifically, we measured performance across different genomic region sizes (1–100 kb), variant densities (0–20 variants/kb), phasing modes, and cohort sizes (1–2000 samples). Overall, CRISPR-HAWK exhibited robust and consistent performance, maintaining scalability across all tested conditions without marked degradation. Comprehensive benchmarking results, including detailed runtime scaling with respect to each parameter and dataset size, are provided in Supplementary Data Section 1.

All analyses presented in this paper were performed on a Linux workstation with an AMD Ryzen Threadripper 3970X CPU with 32 cores and 64 GB of RAM.

### 3.1 Variant-aware guide design on the BCL11A +58 erythroid enhancer

To demonstrate CRISPR-HAWK’s functionality, we focused on the BCL11A +58 erythroid enhancer, a regulatory region critical for hemoglobin gene expression (Bauer *et al.* 2013), targeted by the clinically optimized SpCas9 guide sg1617 (Canver *et al.* 2015, Wu *et al.* 2019, Frangoul *et al.* 2021). Analyzing the enhancer region (Fig. 3A), CRISPR-HAWK identified several gRNA candidates on both the reference and alternative genomes (Fig. 3B). Specifically, the tool found 295 gRNAs from 1000G and 268 from HGDP, with 40.3% and 34.3% containing genetic variants, respectively. A small subset (4.07% in 1000G; 2.61% in HGDP) originated from novel PAM motifs introduced by genetic variants and appeared exclusively in non-reference haplotypes. In contrast, the broader variant spectrum of gnomAD yielded a markedly higher fraction of guides carrying variants (94.28%), underscoring the importance of large-scale variant databases for comprehensive guide design (Fig. 3B). Interestingly, using gnomAD variants CRISPR-HAWK identified four alternative gRNAs for sg1617.

**Figure 3:**
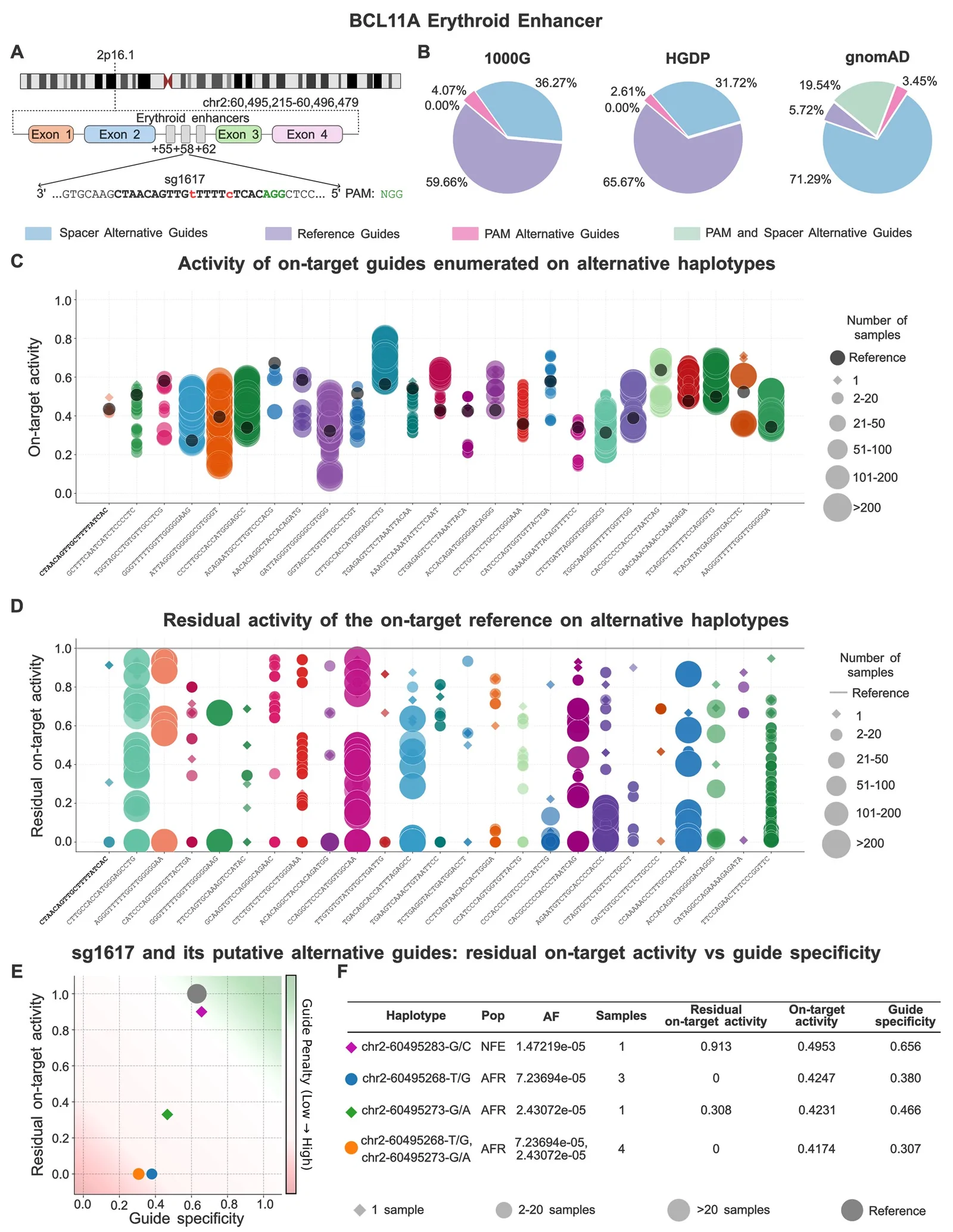
Variant-aware gRNA design and scoring analysis of the BCL11A +58 erythroid enhancer. (A) The clinically optimized guide sg1617 targets the +58 erythroid enhancer located within an intronic region of BCL11A. (B) Classification of gRNAs identified across three variant datasets (1000G, HGDP, and gnomAD): reference guides, spacer alternative guides (variants in spacer only), PAM alternative guides (variants creating novel PAM), and guides with variants in both PAM and spacer. (C) On-target efficiency scores (Azimuth) for the top 25 guides at the BCL11A enhancer, comparing reference sequences to haplotype-matched alternative sequences. Guides are ranked by maximum absolute score difference; sg1617 is shown in bold. Point size indicates the number of individuals carrying each variant. (D) Residual on-target activity (CFD) for the top 25 reference-designed guides at the BCL11A enhancer, showing predicted cleavage efficiency on variant-containing target sequences. Guides are ranked by maximum delta between reference and variant targets; sg1617 is shown in bold. Point size indicates the number of individuals carrying each variant. (E) Relationship between residual on-target activity and guide specificity for sg1617 and its variant-containing target sequences. Background shading indicates guide penalty, with green representing optimal combinations of high residual activity and high specificity. Off-target searches were performed with CRISPRme v2.1.7 using 1000G and HGDP variants, allowing up to six mismatches and two DNA/RNA bulges. (F) Summary of variant-containing target sequences for sg1617, reporting haplotype, population of origin (Pop), allele frequency (AF), sample count, residual on-target activity, on-target efficiency, and guide specificity.

We first assessed the on-target efficiency of guides designed to match specific haplotypes. By comparing the on-target efficiency scores predicted using the Azimuth model across the top 25 guides showing the largest score differences, we observed that specific variant combinations altered predicted activity, either enhancing or reducing efficiency depending on local sequence context (Fig. 3C). Notably, a subset of alleles reduced predicted activity below 0.2, suggesting potential functional impairment. For sg1617 specifically, guides redesigned to perfectly match variant-containing sequences showed Azimuth scores comparable to the reference guide, indicating that variants in this region do not inherently compromise guide design potential. Although these predictions derive from computational models and may not fully reflect in vivo performance, they illustrate how genetic variants can modulate gRNA cutting efficiency. Dataset-specific efficiency estimates using Azimuth score for the BCL11A enhancer are provided in Supplementary Data Section 4 and Fig. S2.

The preceding analysis assumes guides are redesigned to match each patient’s haplotype; however, in clinical practice, a single reference-designed guide is typically administered to all patients. To assess how variants affect the residual on-target activity of reference-designed guides, we computed CFD scores between each guide and its variant-containing target sequences (Fig. 3D). In this context, variants function analogously to mismatches at off-target sites, potentially reducing or abolishing cleavage. Among the top 25 guides showing the largest CFD deltas, we observed a consistent reduction in predicted cleavage efficiency. Several alternative on-target sites displayed complete loss of activity (CFD = 0). Notably, this includes sg1617, the clinically deployed guide, which showed complete loss of predicted activity (CFD = 0) for two target sequences harboring gnomAD variants, suggesting that the approved therapy may have reduced or absent efficacy in patients carrying these variants.

Many of these variants occur at moderate to high population frequencies, implying that guides designed exclusively on the reference genome may fail to achieve effective editing in certain individuals. As with Azimuth, low CFD values do not imply total loss of cleavage but rather a reduced likelihood of efficient binding or cutting. Dataset-specific CFD results are detailed in Supplementary Data Section 5 and Fig. S3.

To complete the characterization, we assessed the off-target potential of sg1617 and its alternatives using CRISPRme v2.1.7 (Cancellieri *et al.* 2023), integrating 1000G and HGDP variants and allowing up to six mismatches and two RNA/DNA bulges (Fig. 3E and F). Among the alternative target sequences (Fig. 3F), those carrying the haplotypes chr2-60495268-T/G-chr2-60495273-G/A and chr2-60495276-G/A exhibited complete loss of predicted cleavage activity (CFD = 0), whereas the sequence carrying haplotype chr2-60495273-G/A retained moderate activity (CFD = 0.308). In contrast, the alternative target with haplotype chr2-60495283-G/C showed minimal impact on residual on-target activity (CFD = 0.913). Regarding guide specificity, most alternative sequences for sg1617 displayed a modest improvement in predicted specificity (Fig. 3E). Notably, haplotype chr2-60495283-G/C maintained both high residual on-target activity (CFD = 0.913) and specificity comparable to the reference guide, whereas haplotypes with reduced on-target activity (CFD = 0) showed improved specificity, likely because the same mismatches that reduce on-target cleavage also reduce off-target binding.

To generalize these findings, we extended the analysis to two loci targeted by the CRISPR-Cas12a system, HBG1 and HBG2 (De Dreuzy *et al.* 2019). The analysis revealed similar patterns to those observed for BCL11A, where genetic variation modulated guide performance (Supplementary Data Section 6 and Figs. S4 and S5).

Taken together, these results demonstrate that CRISPR-HAWK enables comprehensive assessment of guide performance across three dimensions: on-target efficiency for haplotype-matched designs, residual activity when reference guides encounter variant targets, and genome-wide specificity.

### 3.2 Evaluation of on-target activity of BCL11A +58 erythroid enhancer using multiple scoring models

Because CRISPR-HAWK reports on-target efficiency predictions from multiple models rather than a single consensus score (Section 2.4.1), we assessed the concordance among these independent estimates for haplotype-resolved guides. We scored all gRNAs identified at the BCL11A +58 erythroid enhancer using the five SpCas9 predictors supported by CRISPR-HAWK, namely Azimuth (Doench *et al.* 2016), RS3 (DeWeirdt *et al.* 2022), CRISPRon (Anthon *et al.* 2022), PLM-CRISPR (Hou *et al.* 2025), and sgDesigner (Hiranniramol *et al.* 2020), across sequences enriched with variants from the 1000G, HGDP, and gnomAD datasets (Fig. 4). Predicted efficiencies varied across all predictors, indicating that sequence variation can markedly affect predicted editing activity irrespective of the scoring framework. Despite these differences in absolute predictions, the methods showed agreement in ranking high-performing candidates. Among the top-ranked guides, Azimuth (Fig. 4A) shared five candidates with both RS3 (Fig. 4B) and CRISPRon (Fig. 4C), and six candidates with PLM-CRISPR (Fig. 4D). sgDesigner (Fig. 4E) showed the lowest overlap with the other predictors, with two to four shared guides, consistent with its distinct feature set and plasmid-based training data. Across all guides identified within the enhancer, pairwise Pearson correlation coefficients ranged from 0.58 to 0.87 (Fig. 4F), indicating moderate-to-high concordance between methods. Higher correlations were generally observed between models with similar feature representations, whereas lower correlations reflected differences in model assumptions and training data. Overall, the moderate-to-high concordance between predictors supports the use of multiple scoring models in CRISPR-HAWK, which reports each prediction independently allowing users to select the most appropriate model.

**Figure 4:**
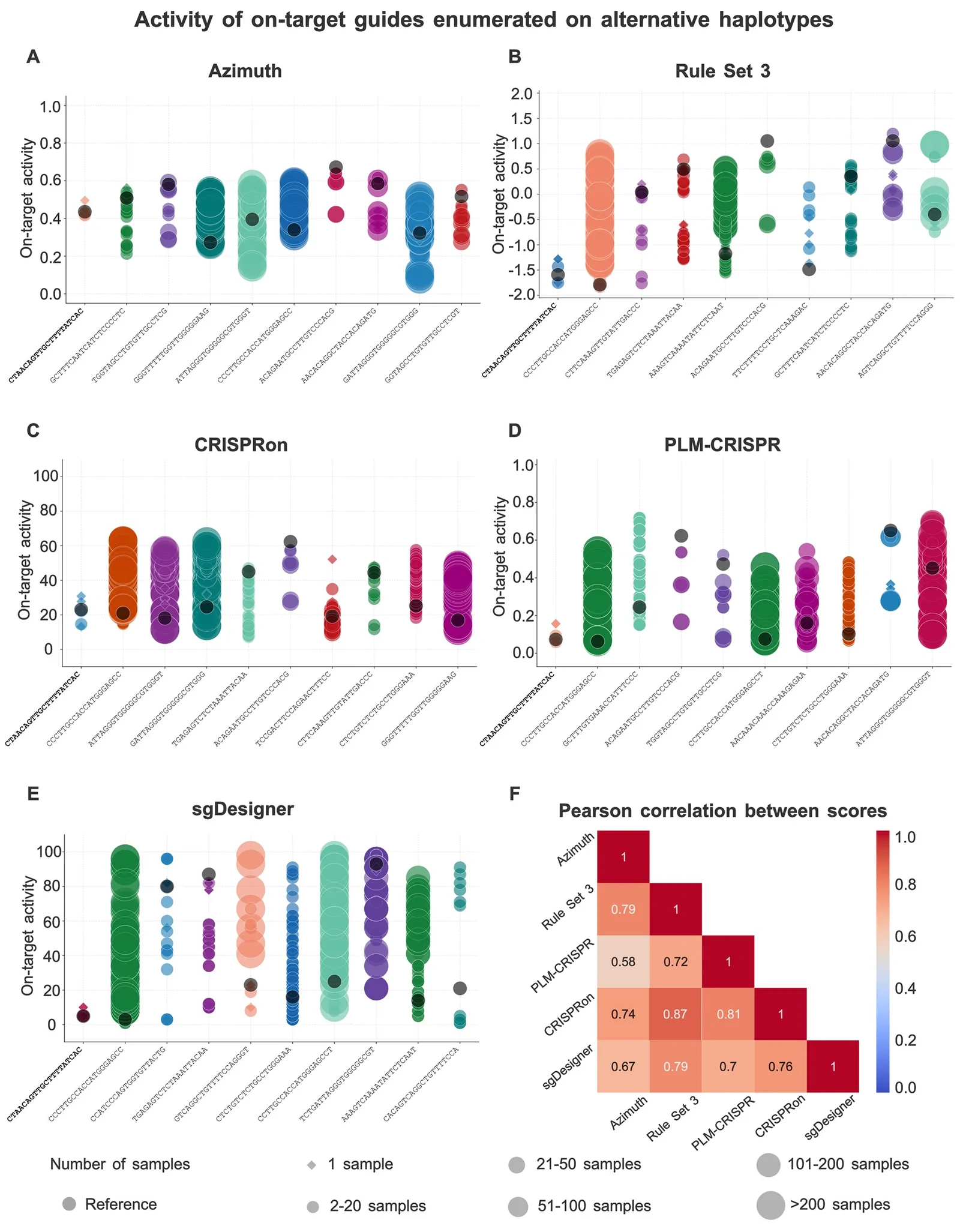
On-target activity scores are computed using Azimuth (A), Rule Set 3 (B), CRISPRon (C), PLM-CRISPR (D), and sgDesigner (E), using genetic variants from the 1000 Genomes Project, HGDP, and gnomAD datasets. Each point represents a guide RNA; point size reflects the number of samples carrying the corresponding variant(s), while diamonds denote singleton gRNAs (variant guides carried by single individuals). Reference guides are shown in dark, whereas haplotype-matched guides are color-coded. For each model, the top 10 guides ranked by the maximum absolute difference in predicted efficiency between reference and alternative haplotypes are displayed. (F) Heatmap of pairwise Pearson correlation coefficients between efficiency scores computed across all guides identified within the BCL11A enhancer region.

### 3.3 Residual on-target activity of therapeutic and benchmark gRNAs

We next extended the residual on-target activity analysis to additional genomic loci targeted by gRNAs in clinical or preclinical development (Fig. 5A) including TRBC1/TRBC2 (Stadtmauer *et al.* 2020), HBB (DeWitt *et al.* 2016, Xu *et al.* 2019b), HBG1/HBG2 (De Dreuzy *et al.* 2019, Métais *et al.* 2019) and CCR5 (Xu *et al.* 2017, 2019a) genes. To provide broader context, we also included EMX1 and FANCF, benchmark loci commonly employed in off-target assessment studies (Tsai *et al.* 2015). For each guide, we computed CFD scores between the reference-designed sequence and all variant-containing target sequences identified across the three population datasets.

**Figure 5:**
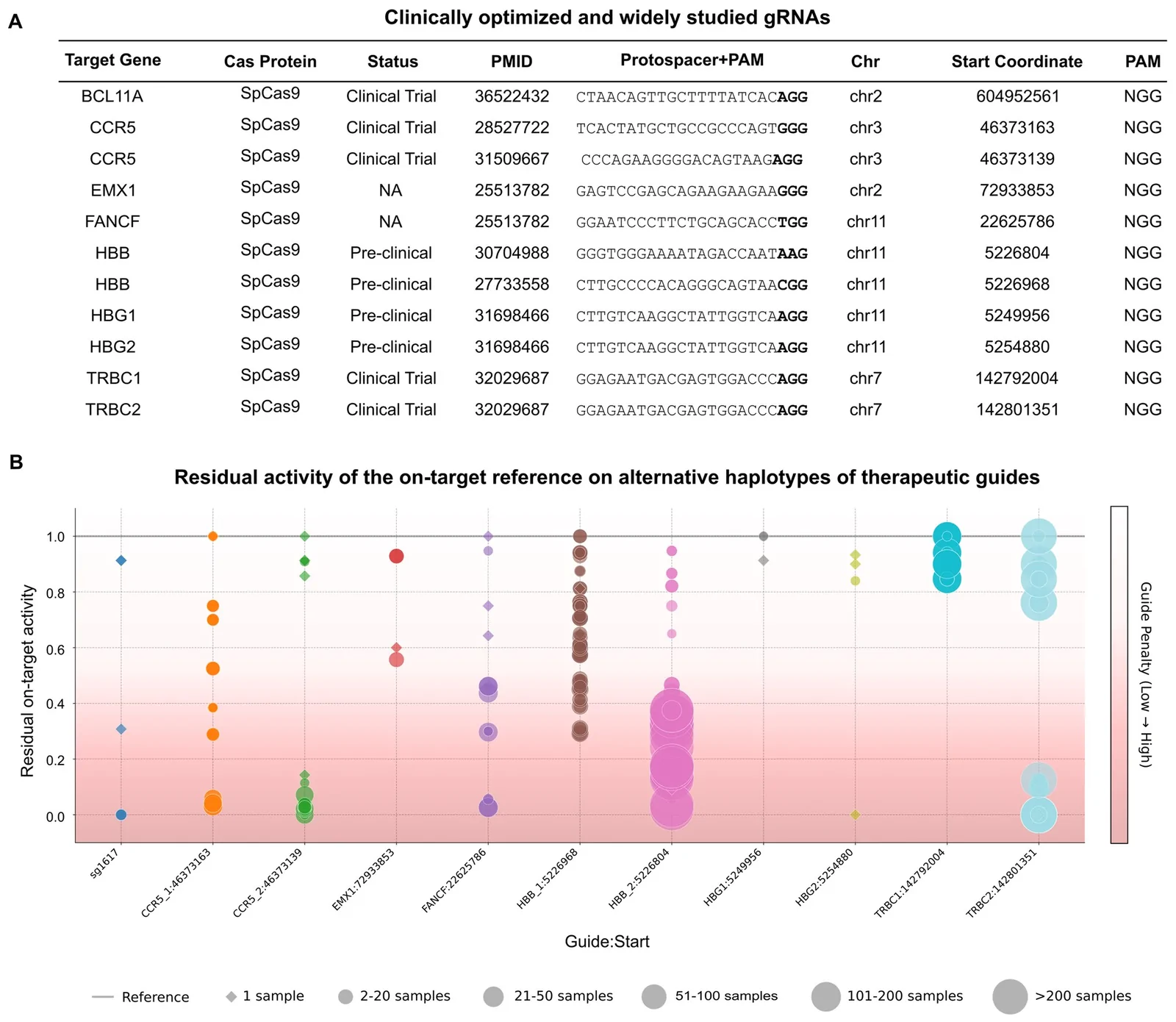
Residual on-target activity of therapeutic and benchmark gRNAs across variant-containing target sites. (A) Summary of analyzed guides, including target genes, Cas nucleases, clinical status, genomic coordinates, and PAM sequences. (B) CFD scores quantifying predicted cleavage efficiency of reference-designed guides on variant-containing target sequences. Each point represents a variant-containing target sequence; point size reflects the number of individuals carrying the corresponding variant(s).

For most analyzed guides, variants reduced predicted cleavage efficiency, with several cases showing a complete loss of activity (CFD = 0) at specific variant-containing target sites (Fig. 5B). Notably, variants causing loss of cleavage activity often occurred in haplotypes shared by more than 200 individuals, as observed for HBB_2:5226804 and TRBC2:142801351 (Fig. 5B). In contrast, other variants modulated gRNA activity more moderately, resulting in only partial decreases in CFD scores and suggesting a lesser impact on cleavage potential. For some guides, such as those targeting TRBC1, all variant-containing sites retained high predicted activity, indicating relatively low susceptibility to sequence variation at this locus.

Across the seven loci analyzed, 82.5% of reference-designed guides contained at least one variant predicted to modify on-target activity. Collectively, these results emphasize that even clinically validated gRNAs designed on the reference genome may experience substantial variability in predicted editing efficiency when applied to genetically diverse populations.

### 3.4 Validating CRISPR-HAWK sgRNA predictions

To further validate CRISPR-HAWK on real genetic variation, we leveraged publicly available population-scale datasets to identify naturally occurring variants. As no public dataset currently combines on-target genome-editing measurements with genotype- or haplotype-resolved information for the same samples, direct experimental validation of on-target prediction is not feasible. For off-target predictions, we previously reported and experimentally validated variant-induced off-target effects for the BCL11A sg1617 guide in Cancellieri *et al.* (2023), and CRISPR-HAWK incorporates this validated framework via its integration with CRISPRme. To complement this with a model-independent assessment of on-target feasibility, we adopted a validation strategy grounded in a deterministic property of CRISPR-Cas systems, namely the strict requirement for an intact PAM. We assessed whether CRISPR-HAWK accurately identifies instances in which variants disrupt the canonical NGG PAM required by SpCas9, thereby abolishing targetability independently of any sequence-based predictive models. We identified multiple PAM-disrupting variants across diverse populations for which CRISPR-HAWK consistently predicts loss of valid targeting sites (see Supplementary Data Section 7 for details). These cases identify individuals in the analyzed cohorts for whom current reference-based therapies are predicted to fail by deterministic argument alone, demonstrating that CRISPR-HAWK delivers clinically actionable findings even in the absence of variant-resolved on-target validation datasets.

## 4 Conclusions

CRISPR-HAWK’s core contribution consists of original engineering work specifically developed to enable variant- and haplotype-aware gRNA design at population scale. While CRISPR-HAWK leverages established off-target search engines (CRISPRitz, CRISPRme) and on-target scoring models, the methodological contributions at its core, namely efficient haplotype reconstruction from VCF data, haplotype-aware guide scanning with binary-encoded PAM matching, and per-nucleotide variant-to-spacer provenance tracking for residual on-target activity computation, are not available in any existing tool and cannot be reproduced through script-level chaining. Reconstructing per-individual haplotypes requires the sequential propagation of SNVs and indels along the target sequence while maintaining nucleotide-resolution coordinate mappings that remain consistent after each insertion or deletion event. In the absence of phasing information, heterozygous variants introduce combinatorial ambiguity that must be resolved efficiently. CRISPR-HAWK addresses this challenge through IUPAC-based encoding combined with selective enumeration restricted to candidate guide sequences, thereby avoiding the combinatorial explosion inherent to naïve approaches, and collapses biologically equivalent haplotypes via hashing-based deduplication to enable efficient processing of large cohorts. Computing residual on-target activity further requires a rigorous, per-nucleotide correspondence between each variant-containing spacer and its reference-derived counterpart, which cannot be reconstructed through post-hoc annotation of standard tool outputs. These components are tightly interdependent. Inaccuracies in indel offset propagation compromise downstream coordinate mappings. Incorrect haplotype deduplication increases computational redundancy, and loss of variant location undermines the interpretability of residual on-target activity scores. By encapsulating these interdependent processes within a unified and validated framework, CRISPR-HAWK enables reproducible and scalable population-aware gRNA design without requiring users to independently implement and validate each computational layer.

In this study, we introduced CRISPR-HAWK, a computational framework that integrates individual- and population-specific genetic variants and haplotype information into CRISPR-Cas gRNA design and evaluation. By accounting for genetic diversity, CRISPR-HAWK enables variant- and haplotype-aware gRNA design and assessment of three complementary performance metrics: on-target efficiency for haplotype-matched guides, residual on-target activity when reference-designed guides encounter variant targets, and genome-wide specificity, supporting the development of population-aware and personalized genome editing strategies.

CRISPR-HAWK’s core contribution consists of original engineering work specifically developed to enable variant- and haplotype-aware gRNA design at population scale. While CRISPR-HAWK leverages established off-target search engines (CRISPRitz, CRISPRme) and on-target scoring models, the methodological contributions at its core, namely efficient haplotype reconstruction from VCF data, haplotype-aware guide scanning with binary-encoded PAM matching, and per-nucleotide variant-to-spacer provenance tracking for residual on-target activity computation, are not available in any existing tool and cannot be reproduced through script-level chaining. Reconstructing per-individual haplotypes requires the sequential propagation of SNVs and indels along the target sequence while maintaining nucleotide-resolution coordinate mappings that remain consistent after each insertion or deletion event. In the absence of phasing information, heterozygous variants introduce combinatorial ambiguity that must be resolved efficiently. CRISPR-HAWK addresses this challenge through IUPAC-based encoding combined with selective enumeration restricted to candidate guide sequences, thereby avoiding the combinatorial explosion inherent to naïve approaches, and collapses biologically equivalent haplotypes via hashing-based deduplication to enable efficient processing of large cohorts. Computing residual on-target activity further requires a rigorous, per-nucleotide correspondence between each variant-containing spacer and its reference-derived counterpart, which cannot be reconstructed through post-hoc annotation of standard tool outputs. These components are tightly interdependent. Inaccuracies in indel offset propagation compromise downstream coordinate mappings. Incorrect haplotype deduplication increases computational redundancy, and loss of variant location undermines the interpretability of residual on-target activity scores. By encapsulating these interdependent processes within a unified and validated framework, CRISPR-HAWK enables reproducible and scalable population-aware gRNA design without requiring users to independently implement and validate each computational layer.

Applying CRISPR-HAWK to the BCL11A +58 erythroid enhancer, we demonstrated that naturally occurring variants can substantially alter gRNA sequence composition and predicted editing performance. Integration of population-scale variant data from 1000 Genomes, HGDP, and gnomAD revealed that a large fraction of candidate guides contains variants capable of modifying PAM recognition or spacer complementarity. Critically, analysis of sg1617, the guide used in Casgevy, revealed that certain variant-containing target sequences are predicted to completely abolish cleavage activity (CFD = 0), suggesting that the approved therapy may have reduced efficacy in individuals carrying these variants. Extending this analysis to therapeutic and benchmark gRNAs across seven loci confirmed these findings: 82.5% of reference-designed guides contained at least one variant predicted to modify on-target activity. These results emphasize the need for variant-aware evaluation in translational genome editing pipelines. Despite characterizing guide performance across these three axes, several limitations should be noted. CRISPR-HAWK relies on computational predictors that, while trained on experimental data, may not fully capture in vivo editing outcomes; experimental validation remains essential for clinical applications. The supported scoring models were developed and validated for specific Cas nucleases (Azimuth and CFD for SpCas9; DeepCpf1 for Cas12a); performance may vary when applied to other enzymes or engineered variants. The current implementation primarily addresses single nucleotide variants, with limited support for complex indels that may also affect guide performance. Additionally, predictions do not account for chromatin accessibility or local epigenetic context, which can substantially influence editing efficiency at specific loci. Delivery efficiency, which varies across cell types and delivery methods, is also beyond the scope of the current framework. The use of CFD and Elevation scores as surrogates for predicting residual on-target activity extends beyond their original scope. Both models were developed to quantify mismatch penalties at off-target genomic sites, and neither has been validated against variant-modified on-target sequences in genotype-resolved experimental or clinical settings. Their application here rests on the assumption that the same mismatch-tolerance mechanisms governing off-target cleavage also govern the reduction in on-target activity caused by sample-specific variants. Such estimates should therefore be interpreted as computational approximations of mismatch tolerance rather than predictors of therapeutic efficacy. Finally, population variant databases, while extensive, may not capture rare or population-specific variants relevant to individual patients.

From a translational perspective, the scoring models implemented in CRISPR-HAWK provide a practical framework for guide RNA selection. The first step is to assess targetability, as guides whose PAM sequence is disrupted by genetic variants are unlikely to support efficient editing and should therefore be excluded. Among the remaining candidates, predicted on-target efficiency, residual on-target activity in variant carriers, and variant-aware specificity provide complementary criteria for prioritization. Allele frequencies and population annotations further help assess the clinical relevance of residual variant sensitivity across different populations. Although the relative importance of these criteria depends on the intended application, prioritizing targetability before optimizing guide performance offers a rational and systematic strategy for both personalized and population-scale genome editing. A detailed description of this workflow is provided in Supplementary Data Section 8.

Overall, CRISPR-HAWK provides a comprehensive and scalable solution for assessing the functional consequences of genetic variation on CRISPR-Cas targeting. Its ability to incorporate haplotype and population-level variant data represents a methodological advancement toward precision genome editing, facilitating the development of guides optimized for diverse genetic backgrounds and enhancing the safety and efficacy of therapeutic interventions.

## Supplementary Material

btag708_Supplementary_Data

## Data Availability

The CRISPR-HAWK source code (v0.1.2) is available at https://github.com/pinellolab/CRISPR-HAWK and https://github.com/InfOmics/CRISPR-HAWK. All data used to perform the analyses and generate the figures presented in this paper, including the CRISPR-HAWK guide reports and the off-target sites identified by CRISPRme for the sg1617 gRNA and its haplotype-matched alternatives, are available at https://doi.org/10.5281/zenodo.18070463. The scripts used to generate the results and figures reported in the manuscript are available at https://github.com/pinellolab/CRISPR-HAWK/tree/main/paper and at https://github.com/InfOmics/CRISPR-HAWK/tree/main/paper.
