# Supplementary material for: CRISPR-HAWK: haplotype- and variant-aware guide design toolkit for CRISPR-Cas
Source: Bioinformatics. 2026 Oct 14;42(10):btag708. doi: 10.1093/bioinformatics/btag708 (PMC13674355; doi:10.1093/bioinformatics/btag708)
Supplement: Supplementary file 1 [file btag708_supplementary_data.pdf]

Supplementary Data of “CRISPR-HAWK: Haplotype- and Variant-aware guide design toolkit for CRISPR-Cas”

Alisa Kumbara[1,†], Manuel Tognon[2,†], Giulia Carone[2], Arianna Fontanesi[2], Nicola Bombieri[1], Rosalba Giugno[2,*], and Luca Pinello[3,4,5,*]

[1]Department of Engineering for Innovation Medicine, University of Verona, Verona, 37134, VR, Italy, [2]Department of Computer Science, University of Verona, Verona, 37134, VR, Italy, [3]Molecular Pathology Unit, Center for Computational and Integrative Biology and Center for Cancer Research, Massachusetts General Hospital, Charlestown, 02129, MA, USA, [4]Department of Pathology, Harvard Medical School, Boston, 02115, MA, USA and [5]Broad Institute of MIT and Harvard, Cambridge, 02142, MA, USA

† These authors contributed equally.

∗ Corresponding authors

1 CRISPR-HAWK Computational Performance Assessment

To assess the scalability of CRISPR-HAWK and quantify the contribution of its binary-encoded PAM matching strategy to runtime and memory efficiency, we conducted a systematic benchmark covering a range of genomic region sizes, variant densities, phasing modes, and sample counts. All benchmarks were performed on a Linux workstation equipped with an AMD Ryzen ThreadRipper 3970X CPU (32 cores) and 64 GB of RAM.

Scoring steps (on-target efficiency and specificity/residual activity) were excluded from all benchmarks. These steps rely on external model APIs individually integrated within CRISPR-HAWK's pipeline, represent the primary bottleneck of end-to-end runtime during typical analyses, and operate independently of the PAM matching strategy. Consequently, excluding them allows the benchmark to more accurately isolate and evaluate the computational performance of the core guide enumeration and variant-aware PAM matching components in CRISPR-HAWK.

Controlled benchmarking requires variant datasets with precisely defined density and phasing. We generated synthetic VCF files seeding variants on chromosome 2. Variant types were assigned stochastically, with single-nucleotide variants (SNVs) comprising 80% of variants, and insertions and deletions (1-5 bp) each representing 10%. To capture both typical and variant-dense genomic contexts, we systematically varied variant densities across five levels: 0 (reference only), 1, 5, 10, and 20 variants per kilobase. For phasing, separate VCFs were generated either with explicit haplotype phase information (phased) or with unresolved genotypes (unphased), the latter scenario inducing

combinatorial haplotype expansion during gRNA sequence reconstruction. A fixed sample number of 100 was employed for benchmarks investigating the effects of region size and variant density.

CRISPR-HAWK encodes each haplotype using a 4-bit per-base representation supporting standard nucleotides and IUPAC ambiguity codes. This representation enables PAM detection through efficient bitwise AND operations rather than character-level string comparisons. Although both the binary-encoded implementation and a naïve character-based approach share the same asymptotic time complexity, $O(H \cdot L)$, with respect to the number of haplotypes H and the region length L, the binary representation substantially reduces constant factors in practice. In particular, the compact encoding reduces the memory footprint of haplotype storage by approximately 40-50% using a 4-bits-per-base representation of the encoded haplotype array.

To empirically quantify these advantages, we benchmarked the binary-encoded implementation against a naïve character-comparison approach across all combinations of region sizes and variant densities described above (**Supplementary Table 1** and **Supplementary Figure 1A—D**). Memory savings were consistent across all tested conditions. For example, at a region size of 100 kb with phased variants, binary encoding reduced peak memory by ~42% at a variant density of 1 variant per kb (4.39 vs. 7.59 GB) and by ~36% at 20 variants per kb (5.75 vs. 8.95 GB) (**Supplementary Figure 1A**). Runtime improvements were most pronounced at small-to-intermediate scales, reaching up to ~1.7x at 10 kb and 1 variant per kb for phased data. At the highest tested variant density (100 kb / 20 variants per kb), runtimes of the two approaches converged (2,601 s vs. 2,743 s), reflecting a shift in the dominant computational cost from PAM matching to haplotype reconstruction (**Supplementary Figure 1B**). Notably, a density of 20 variants per kb substantially exceeds typical variant frequencies observed in the human genome (Consortium et al., 2015) and was included here primarily as a computational stress test.

Within parameter ranges most relevant for practical gRNA design, namely genomic regions of 1-20 kb with variant densities of 1-5 variants per kb, CRISPR-HAWK completes guide enumeration in under one minute using the binary-encoded implementation, with a peak memory footprint below 2GB. Comparable trends were observed when benchmarking unphased variant datasets, where binary encoding consistently reduced both memory consumption and runtime relative to naïve PAM-matching strategy (**Supplementary Figure 1C—D**). As in the phased case performance convergence between the two methods emerged only at the highest variant densities, where the combinatorial expansion of haplotypes required to reconstruct guides carrying unphased variants becomes the dominant contributor to runtime (**Supplementary Figure 1D**).

We next evaluated how runtime and memory scale with the number of samples in the input VCF. For this analysis, we considered a fixed genomic region of 10 kb with a variant density of 5 variants per kb and varied the number of samples from 1 to 2,000 under both phased and unphased configurations (**Supplementary Table 2** and **Supplementary Figure 1 E—F**).

Runtime increases approximately linearly with sample count. For phased inputs, scaling t from 1 to 1,000 samples resulted in a ~350-fold increase in runtime (0.12 s to 41.59 s), consistent with the

expected $O(H)$ dependence on the number of reconstructed haplotypes (**Supplementary Figure 1F**). Analyses performed on unphased inputs were consistently faster than their phased counterparts at equivalent sample numbers (for example, 1,000 samples required 28.61 s compared to 41.59 s for phased data), reflecting the fact the unphased VCFs typically generate fewer distinct haplotypes per region during sequence reconstruction (**Supplementary Figure 1F**).

Memory consumption remained within practical limits across all tested conditions (**Supplementary Figure 1E**). Even at the largest scale evaluated, 2,000 phased samples for a 10 kb region, peak memory usage reached only 4.04 GB demonstrating that CRISPR-HAWK can efficiently process population-scale variant datasets while maintaining a modest memory footprint.

To complement the synthetic benchmarks, we profiled CRISPR-HAWK on a real genomic locus of clinical relevance: the BCL11A +58 erythroid enhancer, using variant datasets from the 1000 Genomes Project (1000G; 2,579 phased samples) (Consortium et al., 2015; Zheng-Bradley, 2019), the Human Genome Diversity Project (HGDP; 929 unphased samples) (Bergström et al., 2020), and the Genome Aggregation Database (gnomAD) (Karczewski et al., 2020) (**Supplementary Table 3** and **Supplementary Figure 1G—H**).

CRISPR-HAWK processed the 1000G and HGDP datasets in 3.66 s and 0.98 s, respectively, with memory usage remaining below 1 GB of memory in both cases. The analysis of the gnomAD dataset required 10.75 s despite involving fewer nominal samples (populations). This increased runtime is attributable to the substantial higher variant density in gnomAD, which leads to the reconstruction of a larger number of distinct haplotypes for PAM scanning.

Overall, these results demonstrate that CRISPR-HAWK maintains efficient runtime and modest memory requirements when applied to real population-scale datasets, confirming that variant- and haplotype-aware gRNA design can be performed on standard workstation hardware without the need for specialized high-performance computing resources.

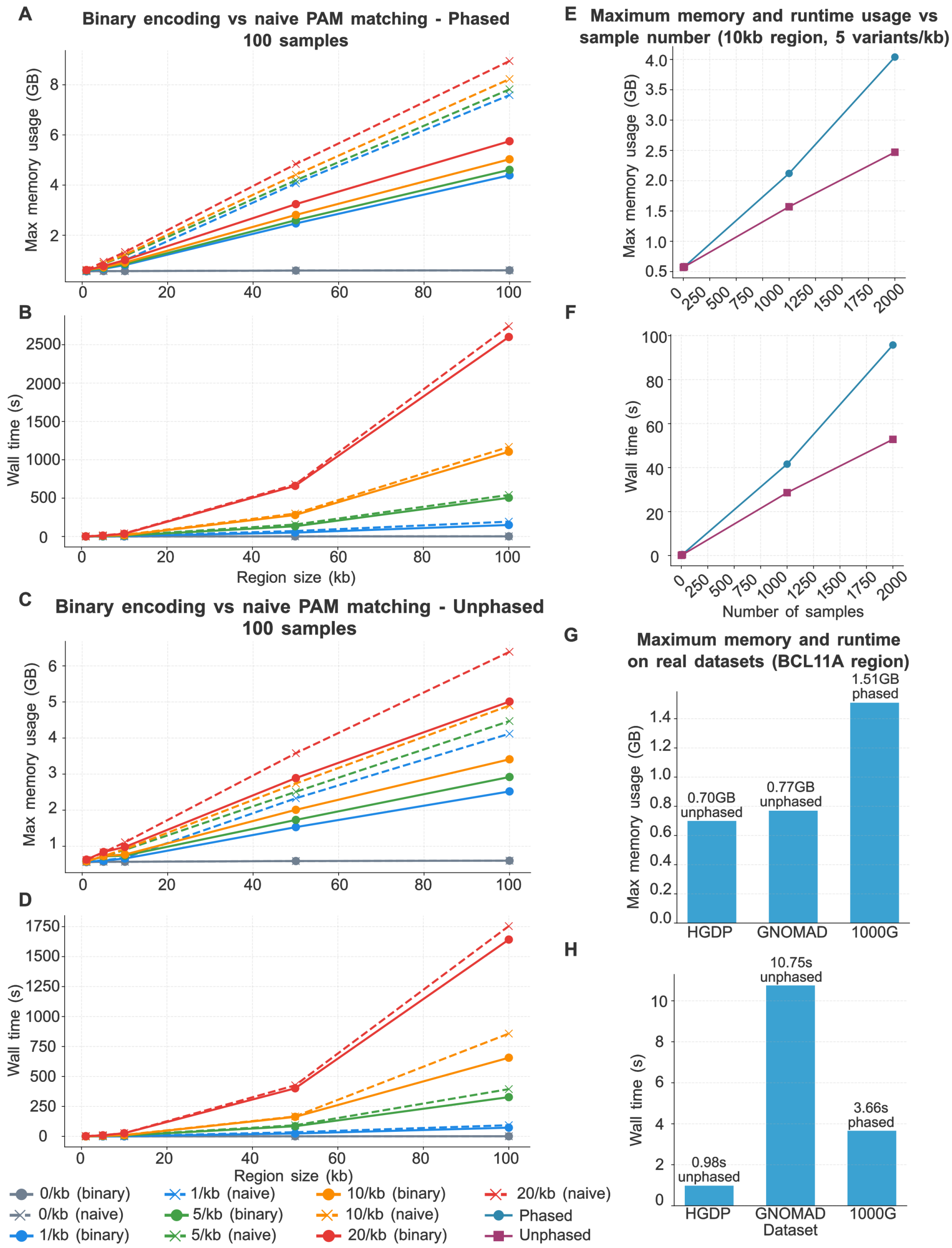

Supplementary Figure 1. Performance benchmarking of CRISPR-HAWK across synthetic and real variant datasets. (A) Maximum memory usage comparison between the binary-encoded PAM matching strategy implemented in CRISPR-HAWK and a naïve character-comparison approach using phased variants (100 samples). **(B)** Wall clock runtime comparison between the binary-encoded PAM matching strategy implemented in CRISPR-HAWK and a naïve character-comparison approach using phased variants (100 samples). Benchmarks were performed across genomic sizes from 1—100 kb and variant densities ranging from 0 to 20 variants per kb. **(C)** Equivalent maximum memory usage benchmark using unphased variants. **(D)** Equivalent wall-clock runtime benchmark using unphased variants. **(E)** Peak memory usage scalability with respect to the number of samples in the input VCF using a fixed 10 kb genomic region with variant density of 5 variants per kb. **(F)** Wall-clock runtime scalability with respect to the number of samples in the input VCF using a fixed 10 kb genomic region with variant density of 5 variants per kb. **(G)** Maximum memory usage profiling on real population-scale datasets at the BCL11A +58 erythroid enhancer using variants from the 1000 Genomes Project, the Human Genome Diversity Project, and the Genome Aggregation Database. **(H)** Wall-clock runtime profiling on real population-scale datasets at the BCL11A +58 erythroid enhancer using variants from the 1000 Genomes Project, the Human Genome Diversity Project, and the Genome Aggregation Database.

Region	Variant-density	Phasing	Time–Binary (s)	Mem–Binary (GB)	Time–Naïve (s)	Mem–Naïve (GB)
1 kb	0/kb	Reference	0.02	0.56	0.03	0.56
1 kb	1/kb	Phased	0.14	0.57	0.14	0.57
1 kb	1/kb	Unphased	0.05	0.57	0.05	0.57
1 kb	5/kb	Phased	0.34	0.59	0.54	0.59
1 kb	5/kb	Unphased	0.19	0.58	0.31	0.58
1 kb	10/kb	Phased	1	0.61	1.56	0.61
1 kb	10/kb	Unphased	0.27	0.58	0.43	0.58
1 kb	20/kb	Phased	1.21	0.61	1.77	0.61
1 kb	20/kb	Unphased	2.29	0.63	2.33	0.62
5 kb	0/kb	Reference	0.07	0.57	0.08	0.57
5 kb	1/kb	Phased	1.91	0.66	3.26	0.72
5 kb	1/kb	Unphased	0.38	0.6	0.51	0.61
5 kb	5/kb	Phased	4.45	0.69	7.01	0.86
5 kb	5/kb	Unphased	3.31	0.72	4.64	0.72
5 kb	10/kb	Phased	8.9	0.72	8.4	0.87
5 kb	10/kb	Unphased	4.35	0.73	5.34	0.73
5 kb	20/kb	Phased	11.6	0.77	13.98	0.94
5 kb	20/kb	Unphased	9.28	0.84	10.03	0.84
10 kb	0/kb	Reference	0.13	0.57	0.25	0.57
10 kb	1/kb	Phased	4.82	0.81	8.19	1
10 kb	1/kb	Unphased	1.71	0.66	2.72	0.68
10 kb	5/kb	Phased	10.66	0.85	15.33	1.18
10 kb	5/kb	Unphased	7.23	0.74	9.52	0.9
10 kb	10/kb	Phased	16.21	0.91	20.74	1.23
10 kb	10/kb	Unphased	10.19	0.77	11.93	0.92
10 kb	20/kb	Phased	35.13	1.01	39.55	1.33
10 kb	20/kb	Unphased	26.78	0.98	27.11	1.11
50 kb	0/kb	Reference	0.67	0.59	0.91	0.59
50 kb	1/kb	Phased	50.5	2.47	70.68	4.07
50 kb	1/kb	Unphased	24.3	1.53	34.94	2.33
50 kb	5/kb	Phased	132.98	2.6	155.79	4.19
50 kb	5/kb	Unphased	84.99	0.98	93.04	2.51
50 kb	10/kb	Phased	279.67	2.81	298.68	4.41
50 kb	10/kb	Unphased	163.16	2.01	166.69	2.74
50 kb	20/kb	Phased	659.83	3.24	678.61	4.84
50 kb	20/kb	Unphased	401.44	2.89	426.28	3.58
100 kb	0/kb	Reference	1.18	0.6	1.53	0.6
100 kb	1/kb	Phased	149.69	4.39	193.11	7.59
100 kb	1/kb	Unphased	72.87	2.52	93.36	4.12
100 kb	5/kb	Phased	504.55	4.61	541.44	7.82
100 kb	5/kb	Unphased	327.74	2.92	394.63	4.47
100 kb	10/kb	Phased	1104.91	5.03	1167.48	8.23
100 kb	10/kb	Unphased	656.52	3.41	857.98	4.9

100 kb	20/kb	Phased	2601.01	5.75	2742.5	8.95
100 kb	20/kb	Unphased	1642.53	5.01	1755	6.39

Supplementary Table 1. Wall-clock time (seconds) and maximum memory usage (GB) for binary encoding and naïve PAM matching phased and unphased synthetic variants. The 0 variants per kb density represent reference-only runs (no VCF). Benchmarks were performed with 100 samples.

Number of samples	Time – Phased (s)	Mem – Phased (GB)	Time – Unphased (s)	Mem – Unphased (GB)
1	0.12	0.57	0.13	0.57
10	0.5	0.58	0.36	0.58
100	10.66	0.85	7.23	0.74
1,000	41.59	2.12	28.61	1.57
2,000	95.74	4.04	52.84	2.47

Supplementary Table 2. Wall-clock time and peak memory as a function of sample count for phased and unphased inputs (10 kb region, 5 variants per kb density, binary encoding).

Dataset	Phasing	Number of samples	Time (s)	Mem (GB)
1000G	Phased	2,579	3.66	0.7
HGDP	Unphased	929	0.98	0.77
gnomAD	Unphased	11*	10.75	1.51

Supplementary Table 3. Wall-clock time and peak memory for CRISPR-HAWK applied to the BCL11A +58 erythroid enhancer with three population-scale variant datasets, using binary-encoded PAM matching (SpCas9 NGG PAM, 20 nucleotides spacer). GnomAD is represented by 11 populations treated as samples. The higher runtime relative to sample number reflects the higher variant density of the gnomAD dataset.

2 Guidance for the Interpretation of On-Target Activity Scores

CRISPR-HAWK reports several on-target activity scores for each candidate guide rather than single efficiency value. This choice reflects a basic property of the prediction task. In fact, On-target activity is an empirical quantity that no model measures directly and the available predictors were trained to approximate it under markedly different conditions. As summarized in **Supplementary Table 4**, the supported models differ in the nuclease and PAM for which they were developed, the length and composition of the guides used for training, the cell types and experimental readouts from which activity was derived, and the machine learning methodology used to relate sequence to activity. Each score therefore encodes a distinct set of biological and experimental assumptions and is best read as a complementary estimate of guide activity rather than as an interchangeable measurement of the same underlying quantity.

A direct consequence is that no single score is universally optimal. A model's predictive accuracy depends on how closely the user's nuclease, guide design, and cellular context resemble the data on which it was trained. Collapsing the individual predictors into a single consensus value would conceal the assumptions on which each score rests, assign comparable weight to models calibrated for unrelated experimental settings, and can yield rankings that appear authoritative yet are difficult to interpret or reproduce. For these reasons, CRISPR-HAWK does not compute a combined or consensus ranking. Instead, every applicable model is run independently and its score reported in parallel, leaving the selection and relative weighting of models to the user.

Selecting an appropriate score begins with the nuclease system, which constitutes a hard compatibility constraint rather than a preference. The scores are not transferable across enzymes. For example, DeepCpf1 (Kim et al., 2018) predicts activity for Cas12a guides and should be used only in that context, whereas the remaining models are intended for SpCas9 guides, with PLM-CRISPR (Hou et al., 2025) additionally accommodating several Cas9 orthologs. **Supplementary Table 4** specifies the nuclease and PAM compatibility of each model.

Within a compatible nuclease, the choice among models should be guided by the experimental context. Because the supported predictors were trained on different guide lengths and scaffolds, in different cell systems, and against different definitions of activity, the most informative score is generally the one whose training conditions most closely match the intended application. **Supplementary Table 4** reports the training dataset characteristics required to make this experimental design choice.

In practice agreement among several applicable models can be interpreted as added confidence in guide's predicted activity, whereas a guide favored by only one model requires closer inspection of that model's assumptions before prioritization. Such strong disagreement is, however, uncommon. Across guides identified in the BCL11A +58 erythroid enhancer, the SpCas9 models showed moderate-to-high pairwise agreement (Pearson R = 0.58—0.87), indicating that the choice of a particular score is rarely decisive for the top-ranked guides. This analysis is detailed in Section **3.2** of the main manuscript. Where scores do diverge, users are advised to favor the model whose training and experimental context best match their application rather than to average across models. Finally, all scores are *in silico* estimates. Therefore, a low predicted value reduces but does not categorically exclude measurable

editing, and the scores are most appropriately used to prioritize candidates for experimental validation, rather than to make absolute accept-or-reject decisions.

Method	Nuclease system	Model architecture	Training dataset description	Publication year
Azimuth	SpCas9, NGG	GBRT	2,500 sequences of 30nt; knockout/loss-of-function screen	2016
Rule Set 3	SpCas9, NGG	GBRT	46,526 sequences of 30nt + tracrRNA; knockout	2022
sgDesigner	SpCas9, NGG	GBRT+ SVM	12,472 sequences of 53nt + scaffold; plasmid library/editing measured by sequencing	2020
CRISPRon	SpCas9, NGG	CNN	23,902 sequences of 30nt; sgRNA activity measured with indels frequency	2022
PLM-CRISPR	7 Cas9 variants, NGG	Protein-language model + CNN	180,000 sequences of 59nt; sgRNA activity measured across Cas9 variants	2025
DeepCpf1	Cas12a, TTTV	CNN	1,251 sequences of 40nt; sgRNA activity measured with indels frequency + chromatin accessibility	2018

Supplementary Table 4. Characteristics and interpretation of on-target activity prediction models integrated in CRISPR-HAWK. The table summarizes the six on-target scoring methods currently supported by CRISPR-HAWK, including their associated nuclease and PAM system, model architecture, training dataset characteristics, experimental setting, and year of publication. This information is intended to guide users in selecting and interpreting the most appropriate score for their nuclease system and experimental context.

Type of score	Method					
Activity of on-target guides on alternative haplotypes	Azimuth	CRISPRon	Deep-Cpf1	Rule Set 3	PLM-CRISPR	sgDesigner
Residual activity on alternative haplotypes	CFD			Elevation		
Guide specificity	CFD			Elevation		

Supplementary Table 5. Scoring methods available in CRISPR-HAWK. Overview of the scoring functions implemented in CRISPR-HAWK, grouped by their functional role. On-target activity across alternative haplotypes is evaluated using multiple predictive models (Azimuth, CRISPRon, DeepCpf1, Rule Set 3, PLM-CRISPR, and sgDesigner). Residual activity on alternative haplotypes and guide specificity are assessed using CFD and Elevation scores, enabling comprehensive evaluation of both efficiency and specificity in a variant-aware context.

3 Repurposing Off-Target Activity Prediction Models to Evaluate Residual On-Target Activity

To quantify the impact of genetic variants on CRISPR-Cas on-target activity, we repurposed established off-target scoring frameworks to measure residual on-target activities. In this formulation, Cutting Frequency Determination (CFD) (Doench et al., 2016) and Elevation (Listgarten et al., 2018) scores are not used for their original purpose (off-target prioritization), but as proxies to estimate how genetic diversity within specific haplotypes or individuals alters the expected activity of an intended target site. This enables a variant-aware assessment of on-target efficiency by translating mismatch- or perturbation-dependent activity penalties into a quantitative reduction of canonical guide performance.

In principle, this formulation is not limited to CFD and Elevation. A broader class of off-target prediction models could be similarly repurposed to estimate variant-dependent perturbations in guide activity. This includes both classical and deep learning-based approaches (Zhang et al., 2023). Early neural architectures such as DeepCRISPR (Chuai et al., 2018) integrate convolutional encoders over guide-target sequences together with epigenetic features, enabling the joint modeling of sequence and chromatin determinants of cleavage activity. More recent models, including CRISPR-Net (Lin et al., 2020) and CRISPR-M (Sun et al., 2024), extend this framework by explicitly representing more complex guide-target interaction structures, such as DNA/RNA bulges and indel-associated alignments, and by leveraging recurrent or attention-based architectures to capture higher-order dependencies. Collectively, these methods demonstrate that off-target scoring functions can be generalized beyond mismatch-only representations to more expressive guide-genome interaction models.

Despite their conceptual applicability, these alternative approaches differ substantially in both formulation and interpretability. DeepCRISPR, for instance, learns a joint representation of sequence and epigenetic context using a convolutional neural network architecture. While it achieves high AUROC values, its improvements over CFD are relatively limited. Gains are more pronounced in AUPRC, indicating improved sensitivity in identifying true off-targets. However, this advantage is not consistently reflected in correlation-based metrics, suggesting limited improvement in capturing continuous variations in cleavage strength across sites.

CRISPR-Net extends DeepCRISPR's paradigm by combining convolutional and recurrent components to better model structured dependencies in guide-target pairing, including bulge configurations. It achieves AUROC values comparable to CFD and Elevation, reflecting similar discriminative power between active and inactive sites, while showing improved AUPRC. CRISPR-M further advances this direction through an attention-based multi-view architectures that enables adaptive weighting of interaction features across mismatch and indel contexts. This results in improved performance over prior models, particularly in datasets enriched for indel-associated off-target events. When restricted to mismatch-only settings, CRISPR-M achieves performance comparable to CFD in AUROC and slightly improved AUPRC.

Among the available scoring strategies, CRISPR-HAWK uses CFD as the basis for residual on-target activity estimation. This choice is motivated by its experimental grounding and demonstrated ability to capture the functional consequences of sequence mismatches on Cas9 activity. Although originally

developed for off-target prediction, CFD has been experimentally validated to accurately reflect the impact of genetic variation on cleavage efficiency, including in variant-aware contexts (Cancellieri et al., 2023). In addition, its explicit mismatch-position and mismatch-type penalty structure provides a transparent and biologically interpretable framework, which is particularly advantageous in large-scale variant-aware analyses where mechanistic interpretability is essential.

Elevation, in contrast, generalizes CFD by aggregating site-level off-target activities into a guide-level specificity score. Rather than modeling individual guide-target interactions in isolation, it integrates multiple CFD-derived estimates into a composite measure of global specificity. While this provides a useful abstraction for guide prioritization, it inherits the underlying assumptions of CFD-based activity modeling and does not fundamentally extend the representation of guide-target biophysics.

Finally, the design modular design of CRISPR-HAWK enables a seamless inclusion of heterogeneous off-target prediction models within a unified computational framework. This design choice ensures that CFD- and Elevation-based residual activity estimates can be naturally complemented or replaced by alternative classical or machine learning-based scoring functions as they mature.

4 Dataset-specific Evaluation of the On-Target Efficiency of BCL11A Enhancer Guides across Variant Haplotypes

By focusing CRISPR-HAWK's analysis on the BCL11A +58 erythroid enhancer (Bauer et al., 2013), targeted by the clinically optimized guide sg1617 (Frangoul et al., 2021; Canver et al., 2015; Wu et al., 2019), we observed that genetic diversity significantly impacts predicted gRNA efficiency. We evaluated activity of on-target gRNAs retrieved across three variant datasets: 1000 Genomes (1000G) (Consortium et al., 2015), Human Genome Diversity Project (HGDP) (Bergström et al., 2020), and Genome Aggregation Database (gnomAD) (Karczewski et al., 2020). Each dataset was used independently to enrich the enhancer region with genetic variants (SNVs and short indels) with different levels of genetic diversity and sample representation.

For each dataset, we identified gRNA candidates on both the reference and alternative genomes. For each candidate guide we predicted its on-target activity using the Azimuth model (Doench et al., 2016). Notably, sg1617 displayed dataset-dependent alternative guide profiles. Enrichment with 1000G (**Supplementary Figure 2A**) and HGDP (**Supplementary Figure 2B**) variants produced no alternative gRNA for sg1617, indicating conservation of its target site in these cohorts. However, adding gnomAD variants (**Supplementary Figure 2C**) revealed four alternative gRNAs exhibiting comparable editing efficiencies, with Azimuth scores between 0.4 and 0.5. This reflects the broader genetic diversity captured by gnomAD, which includes substantially more individuals from a wide range of ancestries.

Beyond sg1617, we observed considerable variability in predicted efficiency among alternative guides. Some reference guides and their variant-derived forms showed similar Azimuth scores, whereas others displayed pronounced shifts in predicted activity depending on their variant content. These differences were consistently larger in the gnomAD dataset than in 1000G or HGDP. Although guides with low predicted efficiency occurred across all databases, the most substantial decreases, sometimes below 0.2, were primarily associated with alternative gRNAs carrying gnomAD variants. Such guides may exhibit reduced or negligible cleavage activity; however, these predictions are based solely on *in silico* modeling. A low Azimuth score does not categorically rule out measurable editing activity in experimental systems.

Overall, these results illustrate that gRNAs designed using a reference genome may show altered, and in some cases improved, performance when optimized for population- or sample-specific genomic contexts. Incorporating individual sequence variation directly into guide design can, under the right conditions, enhance predicted editing efficiency, underscoring the value of variant-aware strategies for personalized therapeutic applications.

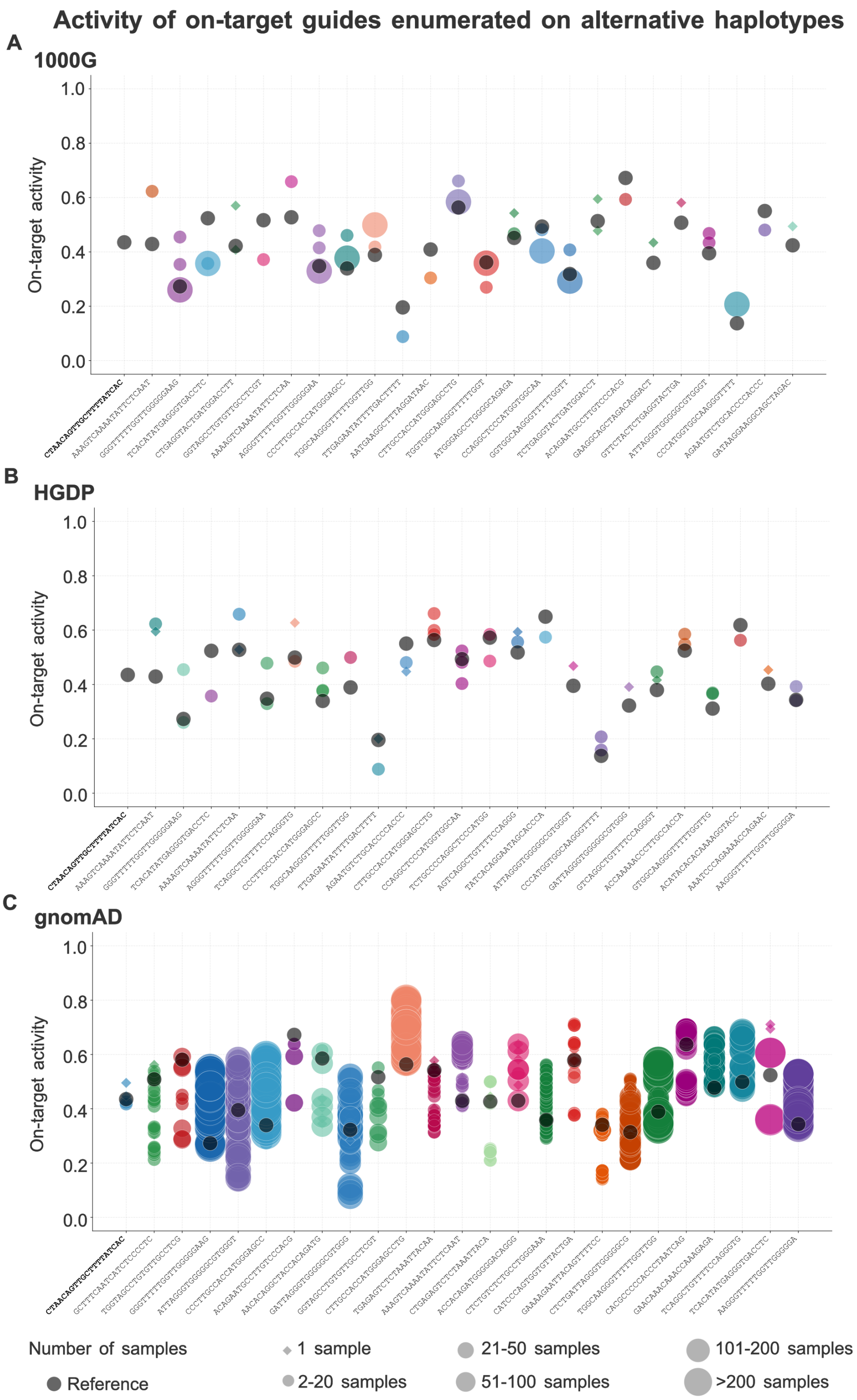

Supplementary Figure 2. On-target efficiency of haplotype-matched guides across BCL11A enhancer. Analyses are based on variant data from 1000G (A), HGDP (B), and gnomAD (C). Each dot represents a guide, with dot size corresponding to the number of samples carrying the variant/s; diamonds indicate singletons. Grey dots represent reference guides, whereas colored dots represent variant-derived guides. The top 25 guides ranked by maximum absolute delta between reference and alternative guides are shown. On-target efficiency is evaluated using the Azimuth model.

5 Dataset-Specific Evaluation of Residual On-Target Activity across Variant Haplotypes

An important aspect in gRNA design is the extent to which guide performance deteriorates when applied to genetically diverse samples, particularly when variants fall within or near the target site. To assess this effect, we computed Cutting Frequency Determination (CFD) scores (Doench et al., 2016) between each reference guide and its corresponding alternative on-target sites within the BCL11A enhancer, independently enriched with variants from 1000G (**Supplementary Figure 3A**), HGDP (**Supplementary Figure 3B**), and gnomAD (**Supplementary Figure 3C**) datasets.

Across all datasets, we observed a general reduction in CFD scores for the top 25 guides exhibiting the largest discrepancies between reference and alternative targets. The magnitude of this reduction showed clear dataset-specific trends. When variant information from 1000G and HGDP was incorporated, most alternative targets maintained moderate CFD scores, typically above 0.4, indicating largely preserved binding and cleavage potential. In contrast, enrichment with gnomAD variants produced not only a higher number of alternative targets but also a substantially larger fraction with markedly reduced CFD scores. Many gnomAD-derived alternative on-target sites fell below 0.5, and several exhibited complete loss of predicted activity (CFD = 0), consistent with a potential abolition of on-target cleavage.

Of particular clinical relevance, two alternative on-target sites for the clinically optimized guide sg1617, identified exclusively using gnomAD variants, displayed CFD scores of zero, suggesting strong disruption of cleavage efficiency in those specific haplotypes. Conversely, another sg1617 alternative site showed no appreciable reduction in predicted activity, illustrating the heterogeneous impact of variation even within the same target region.

As with Azimuth predictions, CFD scores are generated by computational models; therefore, a predicted loss of activity does not definitively imply complete absence of cleavage in biological experiments. Nonetheless, these results highlight the importance of accounting for individual genetic variation. CRISPR-HAWK's ability to systematically detect alternative target sites and quantify the predicted impact of variants provides users with a comprehensive and personalized assessment of guide performance across diverse genomic backgrounds.

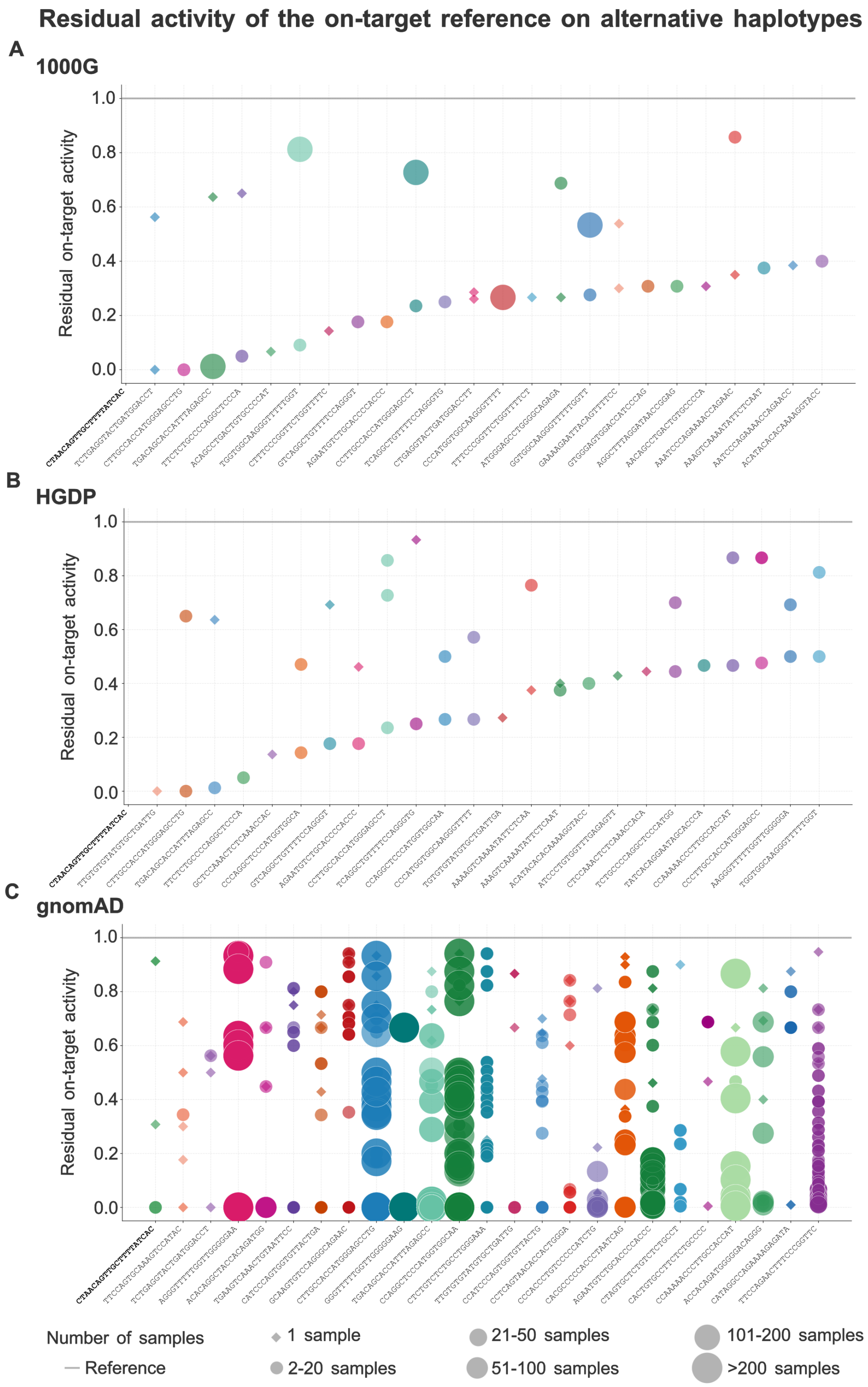

Supplementary Figure 3. Residual on-target activity of haplotype-matched guides across BCL11A enhancer. Analyses are based on variant data from 1000G (A), HGDP (B), and gnomAD (C). Each dot represents a guide, with dot size corresponding to the number of samples carrying the variant; diamonds indicate singletons. The grey line represents reference guides, whereas colored dots represent variant-derived guides. The top 25 guides ranked by maximum delta between reference and alternative guides are shown. Variant effects on alternative on-targets are quantified using the CFD score.

6 Extraction of gRNAs and guide performance analysis for HBG1 and HBG2 genes targeted by CRISPR-Cas12a

To broaden our analysis beyond Cas9 systems, we applied variant-aware guide design and evaluation to the HBG1 (**Supplementary Figure 4**) and HBG2 (**Supplementary Figure 5**) loci (De Dreuzy et al., 2019), both targeted by CRISPR-Cas12a in therapeutic strategies aimed at inducing fetal hemoglobin expression. Retrieved gRNAs were assessed using variants from the 1000G, HGDP, and gnomAD datasets, which were integrated to enrich the DNA sequences of the target regions. To evaluate the contribution of different variant datasets to Cas12a guide design, gRNAs were categorized according to their variant content and database of origin (**Supplementary Figures 5A** and **6A**).

For HBG1, CRISPR-HAWK identified 75 gRNAs from 1000G and 123 from HGDP, with 49.33% and 69.11% containing genetic variants, respectively. Notably, 2.66% of 1000G-derived guides and 8.94% of HGDP-derived guides originated from novel PAM motifs introduced by variants, representing sequences exclusive to non-reference haplotypes that would be missed in conventional reference-based design. For HBG2, 148 gRNAs were retrieved from 1000G and 241 from HGDP, with 45.95% and 66.80% harboring variants, respectively; 3.38% of 1000G and 7.88% of HGDP guides corresponded to variant-induced PAM motifs unique to alternative haplotypes. These proportions mirror those observed for HBG1, indicating similar patterns of variant impact across these closely related loci.

Analysis of the larger and more genetically heterogeneous gnomAD dataset revealed an even higher fraction of variant-containing gRNA candidates for both genes, with over 93% of retrieved gRNAs affected by genetic variation. Predicted on-target efficiencies were estimated using the DeepCpf1 model (Kim et al., 2018), a deep learning framework optimized for Cas12a activity, and guides were ranked according to the largest absolute difference in score between each reference guide and its variant-derived alternatives (**Supplementary Figures 5B** and **6B**). The clinically optimized gRNA and its variant counterparts showed comparable predicted efficiencies, with DeepCpf1 scores ranging between 40 and 70 for both HBG1 and HBG2. Consistent with the BCL11A analysis, variant-derived guides exhibited both increases and decreases in predicted activity relative to reference-based designs, depending on the specific variant content. The magnitude and direction of these efficiency changes aligned with observations from Cas9-targeted regions.

Collectively, these results highlight the importance of incorporating population-specific variants in therapeutic gRNA design, as the majority of potential gRNAs deviate from the reference genome. The concordance of these findings across both HBG loci and across distinct CRISPR systems (Cas9 and Cas12a) reinforces the broad utility of variant-aware strategies in precision genome editing.

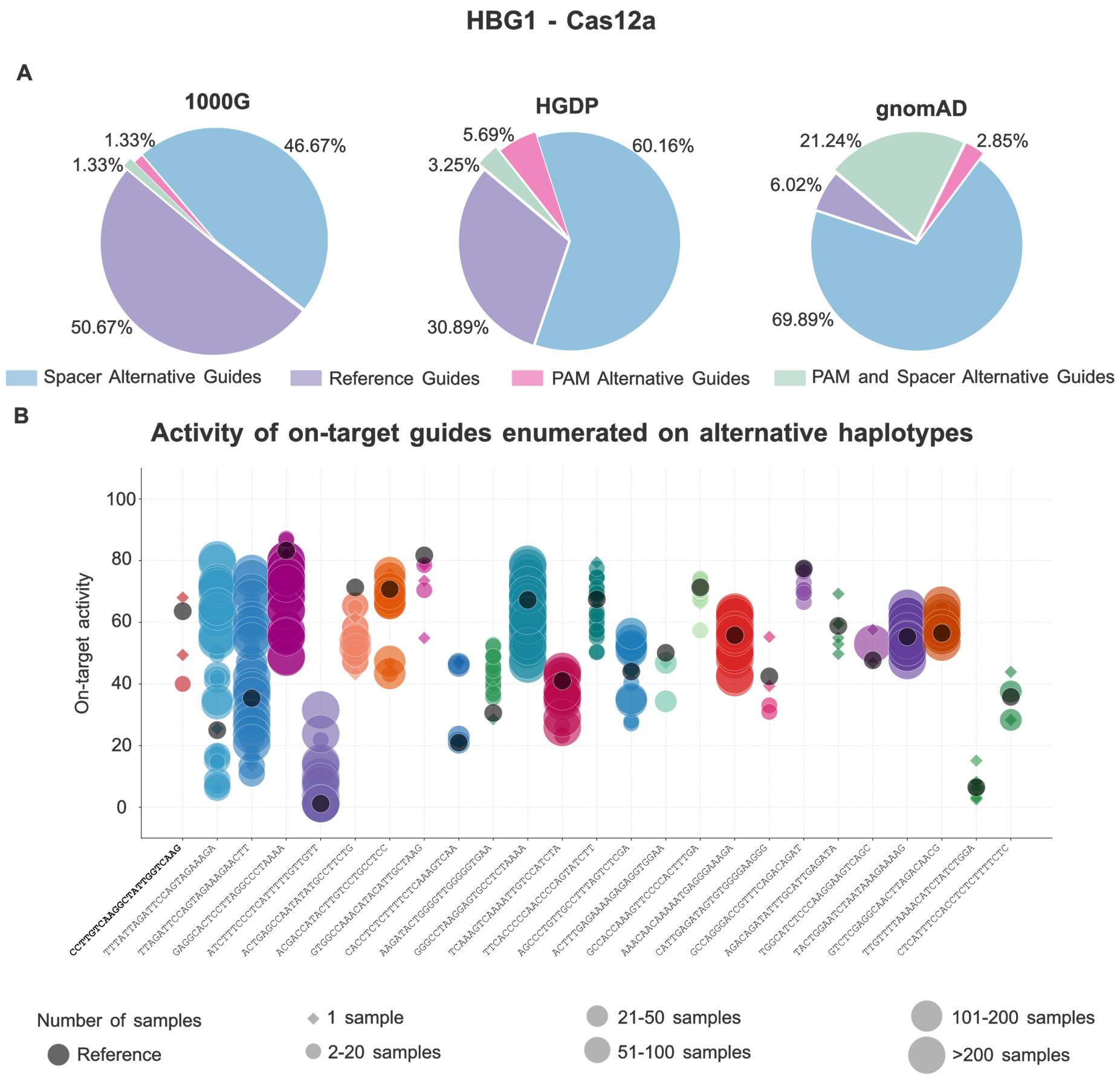

Supplementary Figure 4. Variant-aware gRNA search and scoring analysis of a 2000 bp region of HBG1 gene centered on the therapeutic guide TTTGCCTTGTCAAGGCTATTGGTC. (A) Classification of retrieved guides across three variant datasets (1000G, HGDP, and gnomAD) showing dataset-specific proportions of reference and haplotype-matched guides, distinguishing those containing variants within the PAM, spacer, or both**.** (B) On-target efficiency of reference and haplotype-matched gRNAs across HBG1 evaluated using the DeepCpf1 model. Analyses are based on aggregated variant data from 1000G, HGDP, and gnomAD. Each dot represents a guide, with dot size corresponding to the number of samples carrying the variant/s; diamonds indicate singletons. Grey dots represent reference guides, whereas colored dots represent haplotype-matched guides. The top 25 guides ranked by maximum absolute delta between reference and alternative guides are shown.

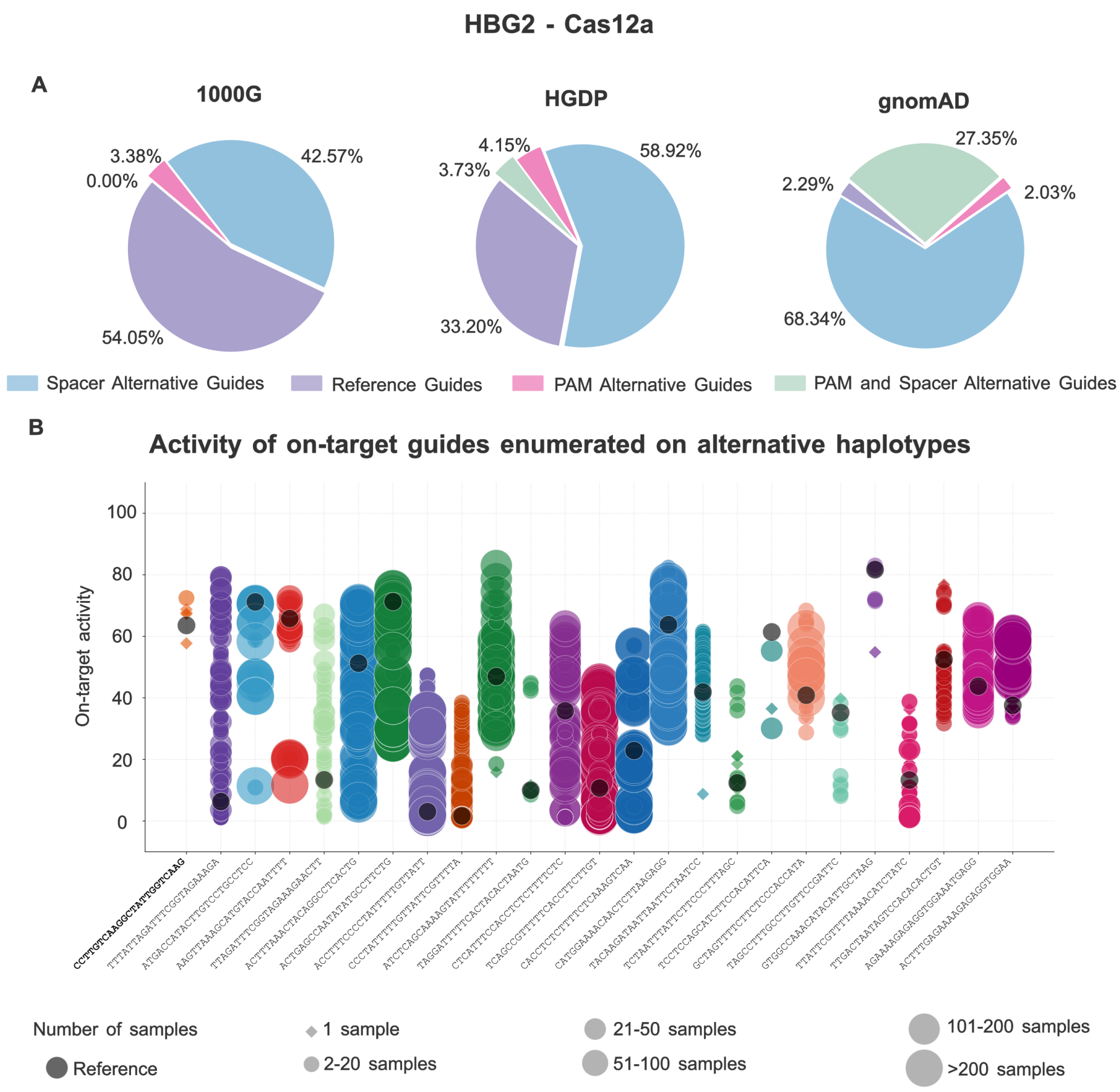

Supplementary Figure 5. Variant-aware gRNA search and scoring analysis of a 2000 bp region of HBG2 gene centered on the therapeutic guide TTTGCCTTGTCAAGGCTATTGGTC. (A) Classification of retrieved guides across three variant datasets (1000G, HGDP, and gnomAD) showing dataset-specific proportions of reference and haplotype-matched guides, distinguishing those containing variants within the PAM, spacer, or both. (B) On-target efficiency of reference and haplotype-matched gRNAs across HBG2 evaluated using the DeepCpf1 model. Analyses are based on aggregated variant data from 1000G, HGDP, and gnomAD. Each dot represents a guide, with dot size corresponding to the number of samples carrying the variant/s; diamonds indicate singletons. Grey dots represent reference guides, whereas colored dots represent haplotype-matched guides. Top 25 guides ranked by maximum absolute delta between reference and alternative guides are shown.

7 Validating CRISPR-HAWK through variant-induced disruption of PAM sites on therapeutic targets

To further support the validity of CRISPR-HAWK predictions, we sought to evaluate its ability to capture variant effects on on-target activity using publicly available genome editing datasets. Ideally, such validation would rely on genome editing experiments performed on samples with known genotype or haplotype information. However, to the best of our knowledge, no publicly available datasets currently provide genome editing outcomes jointly with sufficiently resolved genetic variants at individual or haplotype level for the loci of interest.

In the absence of such data, we designed an alternative validation strategy based on a well-established mechanism and deterministic principle of CRISPR-Cas targeting: the strict requirement for an intact PAM. For SpCas9, disruption of the canonical NGG PAM abolishes nuclease binding and, consequently, editing activity, independently of any predictive model. This provides a robust validation scenario in which the expected outcome is unambiguous.

We therefore investigated whether CRISPR-HAWK can correctly identify cases in which genetic variants disrupt functional PAM sequences at therapeutically relevant and experimentally validated loci (see **Figure 5A** in main manuscript). Specifically, we searched for variants affecting the second or third nucleotide of the NGG motif, which are critical for PAM recognition.

To systematically identify such events, we applied CRISPR-HAWK using a two-step approach. First, we performed a relaxed gRNA search allowing fully degenerate PAM (NNN), thereby identifying all candidate protospacers regardless of PAM compatibility. Second, we compared these results with those obtained under the canonical NGG constraint within the same genomic regions. This comparison enabled the detection of instances in which sequence variation converts a functional NGG PAM into a non-functional motif.

Using this strategy, we identified multiple PAM-disrupting variants for gRNAs targeting CCR5 and HBB supported by population-scale datasets, including gnomAD and the Human Genome Diversity Project. These variants occur in real individuals across diverse populations and, in some cases, are observed in haplotype-resolved samples. For individuals carrying such variants, CRISPR-HAWK correctly predicts the absence of valid targeting sites due to the loss of a functional PAM, implying a complete failure of SpCas9-mediated editing at those loci.

The full list of disrupted PAMs, including genomic coordinates, sgRNA sequences, strand information, GC content, associated variants, allele frequencies, and population annotations, is reported in **Supplementary Table 6**.

This analysis provides a concrete validation of CRISPR-HAWK's ability to capture the functional consequences of genetic variation on genome editing outcomes. Importantly, it demonstrates that the framework accurately identifies variant-induced loss of targetability in real genomic data, even in the absence of direct experimental editing measurements. These results reinforce the importance of

incorporating genotype- and haplotype-aware analyses for the reliable design of CRISPR-based therapeutic strategies.

Chrom	Start	Stop	sgRNA sequence	PAM	Strand	Samples	Variants	AF	Target
3	46,373,139	46,373,162	CCCAGAAGGGGACAGTAAGA	AGc	-	nfe	chr3-46373139-C/G	7e-06	CCR5
11	5,226,804	5,226,827	GGGTGGGAAAATAGACCAAT	AGa	+	afr	chr11-5226826-G/A	2.6e-05	HBB
11	5,226,968	5,226,991	CTTGCCCCACAGGGCAGTAA	CaG	+	afr, asj, eas	chr11-5226989-G/A	2.6e-05	HBB
11	5,226,968	5,226,991	CTTGCCCCACAGGGCAGTAA	CtG	+	sas, HGDP00259	chr11-5226989-G/T	1.08e-03 (HGDP), 1.3e-05 (gnomAD)	HBB
11	5,226,968	5,226,991	CTTGCCCCACAGGGCAGTAA	taG	+	afr	chr11-5226988-C/T, chr11-5226989-G/A	5.9e-05, 2.6e-05	HBB

Supplementary Table 6. Variant-induced disruption of PAM sequences in therapeutically relevant guides targeting CCR5 and HBB. Genetic variants affecting the second or third nucleotide of the canonical NGG PAM are reported. These substitutions convert functional PAMs into non-canonical motifs, thereby abolishing SpCas9 binding and cleavage. For each candidate guide, we report genomic coordinates, sgRNA sequence, disrupted PAM, strand orientation, populations/samples in which the variant is observed, variant identifier, allele frequency (AF), and associated target. Allele frequencies are derived from gnomAD unless otherwise specified. HGDP frequencies are reported when available.

8 Recommended Workflow for Prioritizing Candidate Guides

CRISPR-HAWK reports multiple complementary metrics describing distinct aspects of guide performance, including targetability (PAM integrity in the presence of genetic variants), predicted on-target efficiency, residual on-target activity on variant-containing target sequences (estimated using CFD (Doench et al., 2016) and Elevation (Listgarten et al., 2018)), variant-aware off-target specificity, and the population frequency and ancestry distribution of variants affecting guide performance. Because these metrics capture different biological properties rather than alternative estimates of the same phenomenon, they should not be collapsed into a single composite score. For example, a guide predicted to be highly efficient cannot compensate for the complete loss of targetability caused by disruption of its PAM sequence. Accordingly, CRISPR-HAWK reports these metrics independently and leaves their integration to the specific biological or clinical application.

For guide prioritization, we recommend beginning by defining the genetic context relevant to the intended application, ranging from an individual genotype for personalized genome editing to population-scale datasets such as 1000 Genomes (Consortium et al., 2015), HGDP (Bergström et al., 2020), or gnomAD (Karczewski et al., 2020) for population-aware guide design. Haplotype reconstruction should precede guide evaluation so that all subsequent analyses are performed on the genetic backgrounds of interest. Candidate guides whose canonical PAM is disrupted by genetic variants should then be excluded, as the loss of a functional PAM abolishes nuclease recognition irrespective of any predicted efficiency score. This situation is exemplified by our analyses of the CCR5 and HBB loci, where common variants convert canonical PAMs into non-functional motifs (**Supplementary Data Section 7** and **Supplementary Table 6**).

Among the remaining targetable guides, priority should be given to candidates predicted to maintain high on-target activity across the reconstructed haplotypes using an appropriate nuclease-specific model. Guides predicted to perform well only on the reference genome should be deprioritized because their activity may not generalize across genetically diverse individuals. Residual on-target activity should then be evaluated by comparing each reference guide with its corresponding variant-containing target sequences using CFD or Elevation. Guides exhibiting substantial reductions in predicted activity across common haplotypes are less suitable for broad application, as illustrated by the therapeutic BCL11A sg1617 guide, for which certain gnomAD-derived haplotypes markedly reduce predicted activity whereas others have little or no effect.

Variant-aware specificity should subsequently be considered, particularly in therapeutic settings where minimizing unintended editing remains a key safety objective. Finally, allele frequencies and population annotations provide an essential framework for interpreting the potential clinical impact of variant-associated changes in guide performance, allowing users to prioritize guides that maintain robust activity across common variants and diverse ancestral groups.

Overall, this workflow identifies guides that remain targetable across the genetic backgrounds of interest, maintain high predicted on-target activity, preserve activity in the presence of common genetic variants, and exhibit acceptable variant-aware specificity. We emphasize that this workflow is intended as a recommended strategy rather than a prescriptive decision framework. The relative importance of on-target efficiency, residual activity, specificity, and population context may be adjusted according to the biological question and intended application, whereas targetability should always be treated as a mandatory prerequisite. Importantly, all the required metrics are computed directly by CRISPR-HAWK, allowing this workflow to be applied without any additional analyses beyond a standard software run.

References

D. E. Bauer, S. C. Kamran, S. Lessard, J. Xu, Y. Fujiwara, C. Lin, Z. Shao, M. C. Canver, E. C. Smith, L. Pinello, et al. An erythroid enhancer of bcl11a subject to genetic variation determines fetal hemoglobin level. Science, 342(6155):253–257, 2013.

A. Bergström, S. A. McCarthy, R. Hui, M. A. Almarri, Q. Ayub, P. Danecek, Y. Chen, S. Felkel, P. Hallast, J. Kamm, et al. Insights into human genetic variation and population history from 929 diverse genomes. Science, 367(6484):eaay5012, 2020.

S. Cancellieri, J. Zeng, L. Y. Lin, M. Tognon, M. A. Nguyen, J. Lin, N. Bombieri, S. A. Maitland, M.-F. Ciuculescu, V. Katta, et al. Human genetic diversity alters off-target outcomes of therapeutic gene editing. Nature Genetics, 55(1):34-43, 2023.

M. C. Canver, E. C. Smith, F. Sher, L. Pinello, N. E. Sanjana, O. Shalem, D. D. Chen, P. G. Schupp, D. S. Vinjamur, S. P. Garcia, et al. Bcl11a enhancer dissection by cas9-mediated in situ saturating mutagenesis. Nature, 527(7577):192–197, 2015.

G. Chuai, H. Ma, J. Yan, M. Chen, N. Hong, D. Xue, C. Zhou, C. Zhu, K. Chen, B. Duan, et al. DeepCRISPR: optimized CRISPR guide RNA design by deep learning. Genome biology, 19(1):80, 2018.

1000 G. P. Consortium et al. A global reference for human genetic variation. Nature, 526(7571):68, 2015.

E. De Dreuzy, J. Heath, J. A. Zuris, P. Sousa, R. Viswanathan, S. Scott, J. Da Silva, T. Ta, S. Capehart, T. Wang, et al. Edit-301: an experimental autologous cell therapy comprising cas12a-rnp modified mpb-cd34+ cells for the potential treatment of scd. Blood, 134:4636, 2019.

P. C. DeWeirdt, A. V. McGee, F. Zheng, I. Nwolah, M. Hegde, and J. G. Doench. Accounting for small variantions in the tracrrna sequence improves sgrna activity predictions for crispr screening. Nature Communications, 13(1):5255, 2022.

J. G. Doench, N. Fusi, M. Sullender, M. Hegde, E. W. Vaimberg, K. F. Donovan, I. Smith, Z. Tothova, C. Wilen, R. Orchard, et al. Optimized sgrna design to maximize activity and minimize off-target effects of crispr-cas9. Nature biotechnology, 34(2): 184–191, 2016.

H. Frangoul, D. Altshuler, M. D. Cappellini, Y.-S. Chen, J. Domm, B. K. Eustace, J. Foell, J. de la Fuente, S. Grupp, R. Handgretinger, et al. Crispr-cas9 gene editing for sickle cell disease and β-thalassemia. New England Journal of Medicine, 384(3):252–260, 2021.

Y. Hou, Y. Li, R. Zheng, F. Zhang, F. Guo, M. Li, and M. Zeng. Leveraging protein language models for cross-variant CRISPR/Cas9 sgRNA activity prediction. Bioinformatics, 41(7):btaf385, 2025.

K. J. Karczewski, L. C. Francioli, G. Tiao, B. B. Cummings, J. Alf˙oldi, Q. Wang, R. L. Collins, K. M. Laricchia, A. Ganna, D. P. Birnbaum, et al. The mutational constraint spectrum quantified from variation in 141,456 humans. Nature, 581(7809): 434–443, 2020.

H. K. Kim, S. Min, M. Song, S. Jung, J. W. Choi, Y. Kim, S. Lee, S. Yoon, and H. H. Kim. Deep learning improves prediction of crispr–cpf1 guide rna activity. Nature biotechnology, 36(3): 239–241, 2018.

J. Lin, Z. Zhang, S. Zhang, J. Chen, and K. C. Wong. CRISPR-net: a recurrent convolutional network quantifies CRISPR off-target activities with mismatches and indels. Advanced science, 7(13):1903562, 2020.

J. Listgarten, M. Weinstein, B. P. Kleinstiver, A. A. Sousa, J. K. Joung, J. Crawford, K. Gao, L. Hoang, M. Elibol, J. G. Doench, et al. Prediction of off-target activities for the end-to-end design of crispr guide rnas. Nature biomedical engineering, 2(1):38–47, 2018.

J. Sun, J. Guo, and J. Liu. CRISPR-M: Predicting sgRNA off-target effect using a multi-view deep learning network. PLoS computational biology, 20(3):e1011972, 2024.

G. Zhang, Y. Luo, X. Dai, and Z. Dai. Benchmarking deep learning methods for predicting CRISPR/Cas9 sgRNA on-and off-target activities. Briefings in Bioinformatics, 24(6):bbad333, 2023.

X. Zheng-Bradley, I. Streeter, S. Fairley, D. Richardson, L. Clarke, P. Flicek, and 1000 G. P. Consortium. Alignment of 1000 genomes project reads to reference assembly grch38. Gigascience, 6(7):gix038, 2017.
